# Supplementary material for: Generative Bayesian modeling to nowcast the effective reproduction number from line list data with missing symptom onset dates
Source: PLoS Comput Biol. 2024 Apr 16;20(4):e1012021. doi: 10.1371/journal.pcbi.1012021 (PMC11051644; doi:10.1371/journal.pcbi.1012021)
Supplement: S1 Appendix — (PDF) [file pcbi.1012021.s001.pdf]

Supporting information: Generative Bayesian modeling to nowcast the effective reproduction number from line list data with missing symptom onset dates

Adrian Lison[1,2*], Sam Abbott[3], Jana Huisman[4], and Tanja Stadler[1,2]

[1]Department of Biosystems Science and Engineering, ETH Zurich, Zurich, Switzerland

[2]SIB Swiss Institute of Bioinformatics, Lausanne, Switzerland

[3]Centre for Mathematical Modelling of Infectious Diseases, London School of Hygiene and Tropical Medicine, London, United Kingdom

[4]Physics of Living Systems, Department of Physics, Massachusetts Institute of Technology, Cambridge, Massachusetts, United States of America

[*]Corresponding author: adrian.lison@bsse.ethz.ch

Contents

A Details of modeling components 3

A.1 Time series smoothing . . . 3

Random walk model ▪ Exponential smoothing ▪ Link functions

A.2 Renewal modeling . . . 5

Effective reproduction number ▪ Seeding of infections ▪ Realized infections ▪ EpiEstim

A.3 Modeling of reporting delays . . . 6

Time-to-event model and reporting effects ▪ Backward delays

A.4 Modeling of missing symptom onset dates . . . 8

B Full model definitions 8

B.1 Model I: R_t estimation . . . 9

B.2 Model II: Truncation adjustment . . . 9

B.3 Model III: Joint truncation adjustment and R_t estimation . . . 10

B.4 Model IV: Missing onset date imputation (backward-delay) . . . 11

B.5 Model V: Joint missing onset date, truncation adjustment, and R_t estimation . . . 12

B.6 Priors . . . 13

C Implementation and estimation **16**

C.1 Implementation details . . . 16

C.2 Sampling . . . 16

C.3 Diagnostics . . . 17

D Comparison on synthetic line list data **17**

D.1 Simulation . . . 17

Simulation of infections ▪ Simulation of line list

D.2 Performance evaluation . . . 21

D.3 Supplementary results on synthetic data . . . 21

E Application to COVID-19 in Switzerland **50**

E.1 Hospitalization line list data . . . 50

E.2 Time-varying epidemiological parameters . . . 50

E.3 Overdispersion . . . 52

References **53**

A Details of modeling components

The nowcasting approaches compared in this work use a varying number of models with different complexity. For instance, a fully stepwise approach for incomplete line list data uses a model for imputation, a model for truncation adjustment, and a model for R_t estimation, while a fully generative approach only fits a single, joint model. At the same time, some models share certain characteristics, such as estimating a reporting delay distribution. In the following, we present these shared characteristics and discuss their modeling in detail.

A.1 Time series smoothing

All of our models include one or several latent, time-varying parameters, for example the effective reproduction number R_t, the expected number of symptom onsets λ_t, or the probability of a symptom onset date to become known α_t. When we do not further decompose these parameters via a mechanistic model, we instead specify a non-parametric time series smoothing prior for them. This allows us to estimate the parameters from observed data while providing some regularization through the smoothing structure of the prior.

A.1.1 Random walk model

In our main analysis, we use the simple and popular random walk model [1–3],

$$f(\theta_t)|\theta_{t-1} \sim N(f(\theta_{t-1}), {\sigma_{f(\theta)}}^2),$$

where θ is a time-varying parameter to estimate and f is a link function, e. g. a log link. We further use diffuse hyperpriors for the intercept θ_1 and the variance ${\sigma_{f(\theta)}}^2$ of the random walk (see Supplement B.6), allowing these to be simultaneously estimated from the data. In the context of nowcasting, where comparatively few data are available towards the present, this means that uncertainty about the most recent values of θ also depends on the estimated variance of the random walk. Intuitively, this reflects that expectations are less certain when an observed process exhibited high variance in the past.

A.1.2 Exponential smoothing

We tested the use of a non-stationary smoothing prior as an alternative to the simple random walk prior for λ_t and R_t in the stepwise approach and for R_t in the generative approach. Here we used an innovations state space model with additive errors that implements an exponential smoothing prior according to Holt's linear trend method with dampening [4]. The time-varying parameter of interest, θ, was modeled recursively as

$$\begin{aligned} f(\theta_t) &= l_{t-1} + \phi' b_{t-1} + \epsilon_t \\ l_t &= l_{t-1} + \phi' b_{t-1} + \alpha' \epsilon_t \\ b_t &= \phi' b_{t-1} + \beta' \epsilon_t \\ \epsilon_t &\sim N(0, {\sigma_{f(\theta)}}^2), \end{aligned}$$

where l_t is the level, b_t the trend, and ϵ_t the additive error at time t, respectively. Moreover, α' is a level smoothing parameter, β' a trend smoothing parameter, and ϕ' a dampening parameter. As before, we use hyperpriors for the intercepts l_1 and b_1 and for the variance ${\sigma_{f(\theta)}}^2$ of the innovations (see Supplement B.6), allowing these to be estimated from the data. We also use weakly informative priors for the smoothing parameters α' and β' (Supplement B.6). Due to identifiability issues, the dampening parameter was fixed at $\phi' = 0.9$, meaning that the strength of the trend halves approximately every week of predicting into the future.

A.1.3 Link functions

We use different link functions for the time-varying parameters. For λ_t in the stepwise truncation adjustment approach, we use a log link, which means that increments have a multiplicative effect on the expected number of symptom onsets, which is in line with an exponential growth model on λ_t, where the increments can be interpreted as instantaneous growth rates. For α_t, we use a logit link, which ensures that $0 < \alpha_t < 1$. For R_t, we use a softplus link [5], i. e. $f(x) = \text{softplus}^{-1}(x)$ with $\text{softplus}(x) = \frac{\log(1+e^{kx})}{k}$, where k is a sharpness parameter. We choose $k = 4$ to obtain a function that behaves effectively like $f(x) = x$ for practically relevant values of R_t while ensuring $R_t > 0$. Therefore, increments have approximately additive effects on R_t, which we slightly prefer as a default smoothing assumption, as it avoids asymmetric uncertainty intervals for R_t towards the present

and allows to specify an easily interpretable prior for the random walk variance. Such a model may for example be derived by assuming additive effects of interventions and behavioral changes [6]. However, if a maximum effective reproduction number can be assumed, a viable alternative may be the use of a generalized logit link [7].

A.2 Renewal modeling

A.2.1 Effective reproduction number

In all our models, R_t specifically refers to the *instantaneous* effective reproduction number, i. e. the expected number of secondary infections an individual would cause if conditions remained as they were at time t [8,9]. It is equal to the expected number of infections at time t divided by the total infectiousness at time t, which depends on previous infections weighted by the generation interval distribution [10]. R_t is thus a measure of the overall per-person transmission at time t, including effects of population immunity as well as interventions and behavioral changes. In renewal modeling, R_t is inferred as a latent parameter of the discrete renewal equation [11]

$$\iota_t = E[I_t] = R_t \sum_{s=1}^{G} \psi_s \, I_{t-s},$$

where I_{t-s} is the number of infections that occurred s days before t, and ψ the generation interval distribution with maximum generation time G.

A.2.2 Seeding of infections

The renewal equation is only properly defined for times $t > G$, since new infections may be generated from previous infections up to G days before. The number of initial infections for $t \leq G$ must therefore be modeled using a non-mechanistic prior (also known as seeding when at the start of a new epidemic). We here use a random walk for ι_t on the log scale, i. e.

$$\log(\iota_t)|\iota_{t-1} \sim N(\log(\iota_{t-1}), {\sigma_{\log(\iota)}}^2),$$

with hyperpriors for the intercept and random walk variance as described in Supplement B.6.

A.2.3 Realized infections

To account for noise in the infection process, we assume that the number of realized infections is Poisson distributed with rate ι_t (given through the renewal equation), i. e. $I_t|\iota_t \sim \text{Poisson}(\iota_t)$. However, discrete latent parameters cannot be sampled using the Hamiltonian MCMC No-U-Turn sampler in Stan. Similar to earlier work [11,12], we thus use the Normal distribution as a continuous approximation of the Poisson distribution, i. e. $I_t|\iota_t \sim \text{Normal}(\iota_t, \sqrt{\iota_t})$.

A.2.4 EpiEstim

The package EpiEstim implements a Bayesian framework by Cori et al. [10] to directly compute R_t estimates from the time series of cases by symptom onset date N_t. Under this framework, using a Gamma(a,b) prior for R_t and a smoothing window of τ days, the posterior distribution of R_t is

$$R_t \sim \text{Gamma}\left(a + \sum_{k=0}^{\tau-1} N_{t-k},\ \frac{1}{\frac{1}{b} + \sum_{k=0}^{\tau-1}\sum_{s=1}^{G} N_{t-k-s}\psi_s}\right).$$

A.3 Modeling of reporting delays

A.3.1 Time-to-event model and reporting effects

We express the reporting delay probabilities $p_{t,d}$ via a discrete time-to-event model [3,13]. That is, we model the so-called hazard of reporting $h_{t,d}$, i. e. the conditional probability of a case with symptom onset on date t to be reported with a delay of d days, given that it is not reported earlier. We use logistic regression to parameterize $h_{t,d}$, i. e.

$$\text{logit}(h_{t,d}) = \gamma_d + z_t^\top \beta + w_{t+d}^\top \eta.$$

Here, γ_d is the logit baseline hazard for delay d. Since γ_d is estimated as an independent parameter, we obtain a non-parametric model for the reporting delay distribution with flexible shape. To account for differences in reporting over time, $z_t^\top \beta$ and $w_{t+d}^\top \eta$ are added as additional predictors. Here, z_t is a vector of covariates dependent on symptom onset date t, and w_{t+d} is a vector of covariates dependent on the report date, which is d days after symptom onset date t. Moreover, β and η are vectors of coefficients to be estimated. This specification implements a proportional hazards model [14] for the reporting delay, where $z_t^\top \beta$ describes effects on the hazard by date of symptom onset, and $w_{t+d}^\top \eta$ describes effects by date of report.

We design z_t to account for changes in the reporting delay of cases over time via a piecewise linear change point model [3], i. e.

$$z_t = (z_{t,1}, z_{t,2}, \ldots, z_{t,n}),\ z_{t,i} = \max(0, \min(iK - (T-t), K)),$$

where the i represent $n = ((T-1) \text{ div } K) + 1$ different change points, spaced at $K = 7$ days distance and indexed backward in time from the present (the first changepoint is K days before the present). Note that as the most distant changepoint is not necessarily preceded by K days, it is instead specified as $z_{t,n} = \max(0, \min((n-1)K + ((T-1) \bmod K) - (T-t), ((T-1) \bmod K)))$.

We design w_{t+d} to indicate different weekdays using dummy encoding, i. e.

$$w_{t+d} = (w_{t+d,1}, w_{t+d,2}, \ldots, w_{t+d,6}),\ w_{t+d,i} = \mathbb{1}_{\text{weekday}(t+d)=i},$$

where $i = 1$ represents Mondays and $i = 6$ represents Saturdays (Sundays represent the intercept). When applying our models to real-world data, public holidays were coded as Sundays.

Given the hazard $h_{t,d}$, the probability for a case with symptom onset on date t to be reported with delay d is [13]

$$p_{t,d} = h_{t,d} \prod_{i=1}^{d} (1 - h_{t,d-i}) = h_{t,d}(1 - \sum_{i=1}^{d} p_{t,d-i}).$$

A.3.2 Backward delays

When modeling backward delays, we use a model analogous to the one for forward delays. For a case with date of report t' and reporting delay d, the backward reporting hazard is then

$$\text{logit}(h^{\leftarrow}_{t',d}) = \gamma_d + z_{t'-d}^{\top} \beta + w_{t'}^{\top} \eta.$$

That is, the effects by date of symptom onset and by date of report are indexed relative to the current date of report. Importantly, if data are left-censored, i. e. only cases with date of symptom onset $t \geq 1$ are available, then the backward delay must only be estimated for $t' > D$, otherwise it will be biased by left-truncation.

A.4 Modeling of missing symptom onset dates

By introducing the time-varying parameter α_t, i. e. the probability of a case to be complete, that is to have a known onset date, we can explicitly distinguish between case counts with known and with missing symptom onset date in our model ($N_{t,d}^{\text{known}}$ and $N_{t,d}^{\text{missing}}$). We here index α_t by the date of symptom onset t, but it is in theory also possible to use the date of report $t' = t + d$ instead. In the latter case (date of report), we would have

$$N_{t,d}^{\text{known}} | \lambda_t, p_{t,d}, \alpha_{t+d} \sim \text{Poisson}(\lambda_t \, p_{t,d} \, \alpha_{t+d}), \quad N_{t,d}^{\text{missing}} | \lambda_t, p_{t,d}, \alpha_{t+d} \sim \text{Poisson}(\lambda_t \, p_{t,d} \, (1 - \alpha_{t+d})).$$

Which version is to be preferred will depend on assumptions about the process leading to missing onset dates. We only expect larger differences when a_t is specified via a more complex time series model which e. g. accounts for weekday effects or trends towards the present.

In the joint model for missing onset dates, truncation adjustment, and R_t estimation as specified in Supplement B.5, only observations C_t^{missing} for $t > D$ can be used. This is because the counts C_t^{missing} with $t \leq D$, potentially include cases with symptom onset before $t = 1$. Ignoring these cases would thus lead to bias from left truncation. If C_t^{missing} with $t \leq D$ should be included as observations as well, it is possible to extend the latent parameters λ, p, and α up to D days further into the past. If λ_t is modeled via a generative renewal model as in Supplement B.5, this means that all corresponding parameters such as I_t, ι_t, and R_t must also be extended further into the past. An important disadvantage of this approach is that these extended parameters are potentially informed by very little data. In particular, the reporting delay probabilities $p_{t,d}$ for $t < 1$ are not directly informed by any observations in the line list, meaning that weekday effects but no trends over time can be modeled for these delays.

B Full model definitions

To realize the different approaches compared in this study, we implemented (I) a model for reproduction number estimation, (II) a model for truncation adjustment, (III) a joint model for truncation adjustment and reproduction number estimation, (IV) a model for missing onset date imputation using backward delays, and (V) a joint model for missing onset date, truncation adjustment, and reproduction number estimation. In the following, we provide concise, formal definitions of each

model. We refer the reader to the main text for detailed explanations of the notation and variables.

B.1 Model I: R_t estimation

Model

$C_t \vert \lambda_t \sim \text{Poisson}(\lambda_t)$ / $\text{NegBin}(\lambda_t, \phi)$	cases by report date
$\lambda_t = \sum_{d=0}^{D} I_{t-d}\, \rho_{t-d}\, p_d$	expected cases
$I_t \vert \iota_t \sim \text{Poisson}(\iota_t)$	realized infections
$\log(\iota_t) \vert \iota_{t-1} \sim N(\log(\iota_{t-1}), {\sigma_{\log(\iota)}}^2) \mid t \leq G$	expected infections (seeding)
$\iota_t = E[I_t] = R_t \sum_{s=1}^{G} \psi_s\, I_{t-s} \mid t > G$	expected infections (renewal)
$\text{softplus}(R_t) \vert R_{t-1} \sim N(\text{softplus}(R_{t-1}), {\sigma_R}^2)$	reproduction number

Fixed parameters

$\rho_t = \rho$	ascertainment proportion
$p = (p_0, p_1, \ldots, p_D)$	reporting delay distribution
$\psi = (\psi_1, \psi_2, \ldots, \tau_G)$	generation time distribution

Parameters with priors (see B.6 for priors)

$\frac{1}{\sqrt{\phi}}$	overdispersion
$\log(\iota_1), \sigma_{\log(\iota)}$	random walk seeding phase
R_1, σ_R	random walk reproduction number

B.2 Model II: Truncation adjustment

Model

$N_{t,d} \vert \lambda_t, p_{t,d}, \phi \sim \text{Poisson}(\lambda_t\, p_{t,d})$ / $\text{NegBin}(\lambda_t\, p_{t,d}, \phi)$	cases by onset date & delay

$$p_{t,d} = h_{t,d} \prod_{i=1}^{d} (1 - h_{t,d-i})$$ reporting delay probabilities

$$\text{logit}(h_{t,d}) = \gamma_d + z_t^\top \beta + w_{t+d}^\top \eta$$ reporting hazards

$$\log(\lambda_t)|\lambda_{t-1} \sim N(\log(\lambda_{t-1}), {\sigma_{\log(\lambda)}}^2)$$ expected onsets

Covariates

$$z_t = (z_{t,1}, z_{t,2}, \ldots, z_{t,n})$$ date of symptom onset covariates

$$w_{t+d} = (w_{t+d,1}, w_{t+d,2}, \ldots, w_{t+d,6})$$ date of report covariates

Parameters with priors (see B.6 for priors)

$$\frac{1}{\sqrt{\phi}}$$ overdispersion

γ_d baseline reporting hazard

β_i, η_i reporting delay effects

$\log(\lambda_1), \sigma_{\log(\lambda)}$ random walk expected onsets

B.3 Model III: Joint truncation adjustment and R_t estimation

Model

$$N_{t,d}|\lambda_t, p_{t,d}, \phi \sim \text{Poisson}(\lambda_t p_{t,d}) \;/\; \text{NegBin}(\lambda_t p_{t,d}, \phi)$$ cases by onset date & delay

$$p_{t,d} = h_{t,d} \prod_{i=1}^{d} (1 - h_{t,d-i})$$ reporting delay probabilities

$$\text{logit}(h_{t,d}) = \gamma_d + z_t^\top \beta + w_{t+d}^\top \eta$$ reporting hazards

$$\lambda_t = \sum_{s=0}^{L} I_{t-s} \rho_{t-s} \tau_s$$ expected symptom onsets

$$I_t|\iota_t \sim \text{Poisson}(\iota_t)$$ realized infections

$$\log(\iota_t)|\iota_{t-1} \sim N(\log(\iota_{t-1}), {\sigma_{\log(\iota)}}^2) \mid t \le G$$ expected infections (seeding)

$$\iota_t = E[I_t] = R_t \sum_{s=1}^{G} \psi_s I_{t-s} \mid t > G$$ expected infections (renewal)

$$\text{softplus}(R_t)|R_{t-1} \sim N(\text{softplus}(R_{t-1}), {\sigma_R}^2)$$ reproduction number

Covariates

$z_t = (z_{t,1}, z_{t,2}, \ldots, z_{t,n})$ — date of symptom onset covariates

$w_{t+d} = (w_{t+d,1}, w_{t+d,2}, \ldots, w_{t+d,6})$ — date of report covariates

Fixed parameters

$\rho_t = \rho$ — ascertainment proportion

$\tau = (\tau_0, \tau_1, \ldots, \tau_L)$ — incubation period distribution

$\psi = (\psi_1, \psi_2, \ldots, \tau_G)$ — generation time distribution

Parameters with priors (see B.6 for priors)

$\frac{1}{\sqrt{\phi}}$ — overdispersion

γ_d — baseline reporting hazard

β_i, η_i — reporting delay effects

$\log(\iota_1), \sigma_{\log(\iota)}$ — random walk seeding phase

R_1, σ_R — random walk reproduction number

B.4 Model IV: Missing onset date imputation (backward-delay)

Model

$(N^{\text{known}}_{t,0}, \ldots, N^{\text{known}}_{t-D,D}) \sim \text{Multinom}(p^{\leftarrow}_{t,0}, \ldots, p^{\leftarrow}_{t,D})$ — complete cases by onset date & delay

$p^{\leftarrow}_{t,d} = h^{\leftarrow}_{t,d} \prod_{i=1}^{d} (1 - h^{\leftarrow}_{t,d-i})$ — backward reporting delay probabilities

$\text{logit}(h^{\leftarrow}_{t',d}) = \gamma_d + z^{\top}_{t'-d}\beta + w^{\top}_{t'}\eta$ — backward reporting hazards

Covariates

$z_t = (z_{t,1}, z_{t,2}, \ldots, z_{t,n})$ — date of symptom onset covariates

$w_{t+d} = (w_{t+d,1}, w_{t+d,2}, \dots, w_{t+d,6})$ date of report covariates

Parameters with priors (see B.6 for priors)

γ_d backward baseline reporting hazard

β_i, η_i backward reporting delay effects

Posterior predictive samples

$$p_{t,d}^{\leftarrow(i)} \sim P\left(p_{t,d}^{\leftarrow}, \theta \,\middle|\, N^{\text{known}}_{\{t,d|t+d\leq T\}}\right)$$

$$d_t^{\leftarrow(i)} \sim \text{Categorical}(p_{t,0}^{\leftarrow}, p_{t,1}^{\leftarrow}, \dots, p_{t,D}^{\leftarrow})$$

B.5 Model V: Joint missing onset date, truncation adjustment, and R_t estimation

Model

$$N_{t,d}^{\text{known}} | \lambda_t, p_{t,d}, \alpha_t \sim \begin{array}{c}\text{Poisson}(\lambda_t\, p_{t,d}\, \alpha_t) \;/\\ \text{NegBin}(\lambda_t\, p_{t,d}\, \alpha_t, \phi)\end{array}$$ complete cases by onset date & delay

$$C_t^{\text{missing}} | \lambda, p, \alpha \sim \begin{array}{c}\text{Poisson}(\lambda_t^{\text{mising}}) \;/\\ \text{NegBin}(\lambda_t^{\text{mising}}, \phi)\end{array}$$ incomplete cases by report date

$$\lambda_t^{\text{mising}} = \sum_{d=0}^{\min(D,t-1)} \lambda_{t-d}\, p_{t-d,d}\, (1-\alpha_{t-d})$$ expected incomplete cases by report date

$$p_{t,d} = h_{t,d} \prod_{i=1}^{d} (1 - h_{t,d-i})$$ reporting delay probabilities

$$\text{logit}(h_{t,d}) = \gamma_d + z_t^\top \beta + w_{t+d}^\top \eta$$ reporting hazards

$$\text{logit}(\alpha_t) | \alpha_{t-1} \sim N(\text{logit}(\alpha_{t-1}), {\sigma_{\text{logit}(\alpha)}}^2)$$ probability of known onset date

$$\lambda_t = \sum_{s=0}^{L} I_{t-s}\, \rho_{t-s}\, \tau_s$$ expected symptom onsets

$$I_t | \iota_t \sim \text{Poisson}(\iota_t)$$ realized infections

$$\log(\iota_t) | \iota_{t-1} \sim N(\log(\iota_{t-1}), {\sigma_{\log(\iota)}}^2) \mid t \leq G$$ expected infections (seeding)

$$\iota_t = E[I_t] = R_t \sum_{s=1}^{G} \psi_s\, I_{t-s} \mid t > G$$ expected infections (renewal)

$$\text{softplus}(R_t) | R_{t-1} \sim N(\text{softplus}(R_{t-1}), {\sigma_R}^2)$$ reproduction number

Covariates

$z_t = (z_{t,1}, z_{t,2}, \dots, z_{t,n})$ date of symptom onset covariates

$w_{t+d} = (w_{t+d,1}, w_{t+d,2}, \dots, w_{t+d,6})$ date of report covariates

Fixed parameters

$\rho_t = \rho$ ascertainment proportion

$\tau = (\tau_0, \tau_1, \dots, \tau_L)$ incubation period distribution

$\psi = (\psi_1, \psi_2, \dots, \psi_G)$ generation time distribution

Parameters with priors (see B.6 for priors)

$\frac{1}{\sqrt{\phi}}$ overdispersion

γ_d baseline reporting hazard

β_i, η_i reporting delay effects

$\text{logit}(\alpha_1), \sigma_{\text{logit}(\alpha)}$ random walk for probability of known onset

$\log(\iota_1), \sigma_{\log(\iota)}$ random walk seeding phase

R_1, σ_R random walk reproduction number

B.6 Priors

Table A provides an overview of the priors used for parameters in different model components. Priors were specified broadly to provide regularization to the estimation without distorting results, and the same priors were used across all models. Note that for the random walk models on time-varying parameters such as λ_t, R_t or α_t, we used a hyperprior that allowed the random walk variance to be simultaneously estimated from the data.

For the baseline hazard in the reporting delay model, we provided a constant, diffuse prior across all delays, corresponding to a piecewise exponential distribution in expectation, but with sufficient uncertainty to accommodate various delay distributions. When modeling symptom onsets in model

II, the prior for the intercept of λ was centered on a rough empirical estimate of the expected number of symptom onsets at $t = 1$, i. e. $\hat{\lambda}_1 = \sum_{d=0}^{D}(N_{1,d}^{\text{known}} + \frac{1}{D}C_{1+d}^{\text{missing}})$. Similarly, for the renewal model used in models I, III, and V, the intercept of infections in the seeding phase was given a prior centered on $\hat{\iota}_1 = \frac{1}{\rho}\hat{\lambda}_1$, with ascertainment proportion ρ. Such use of data in the prior only served to constrain the infection time series to the correct order of magnitude. When modeling the effective reproduction number, the use of a softplus link meant that R_t is essentially described via a random walk on the unit scale. Here we chose a wide variance prior that would theoretically allow fast changes in R_t, such as dropping from 4 to 1 within one week.

Table A. Prior choices for model parameters.

Parameter	Description	Prior	Interpretation	Models
**Symptom onsets and transmission**				
$\log(\lambda_1)$	Intercept for log symptom onsets	$\text{Normal}(\mu = \hat{\lambda}_1, \sigma = 0.5)$	Empirical estimate of number of cases with $\times 3$ uncertainty margin	II
$\sigma_{\log(\lambda)}$	Standard deviation of random walk on $\log(\lambda_t)$	$\text{Normal}^+(\mu = \frac{1}{20}, \sigma = \frac{1}{40})$	Daily relative change in cases between $\pm 20\%$	II
$\log(\iota_1)$	Intercept for log infections in seeding phase	$\text{Normal}(\mu = \hat{\iota}_1, \sigma = 0.5)$	Empirical estimate of number of infections with $\times 3$ uncertainty margin	I, III, V
$\sigma_{\log(\iota)}$	Standard deviation of random walk on $\log(\iota_t)$	$\text{Normal}^+(\mu = \frac{1}{20}, \sigma = \frac{1}{40})$	Daily relative change in infections between $\pm 20\%$	I, III, V
R_1	Intercept for effective reproduction number	$\text{Normal}(\mu = 1, \sigma = 0.8)$	Reproduction number initially between 0 and 3	I, III, V
σ_R	Standard deviation of random walk on R_t	$\text{Normal}^+(\mu = 0, \sigma = 0.1)$	Weekly absolute increase or decrease of R_t between ± 3	I, III, V
**Reporting delays**				
γ_d	Baseline logit reporting hazard for a delay of d days	$\text{Normal}(\mu = \mu_\gamma, \sigma = \sigma_\gamma)$, $\mu_\gamma = \text{logit}(1 - 0.01^{1/D})$, $\sigma_\gamma = \frac{1}{2}(\text{logit}(0.98) - \mu_\gamma)$	Piecewise exponential reporting delay with $P(d = 0)$ between 2% and 98% and expectation that $P(d < D) = 99\%$	II, III, IV, V
β_i	Linear trend of the logit reporting hazard during week i	$\text{Normal}(\mu = 0, \sigma = 0.1)$	Odds for daily reporting differ between weeks maximally by a factor of 4	II, III, IV, V
η_i	Effect of weekday i on logit reporting hazard	$\text{Normal}(\mu = 0, \sigma = 0.75)$	Odds for daily reporting differ between weekdays maximally by a factor of 20	II, III, IV, V
**Missing onset dates**				
$\text{logit}(\alpha_1)$	Intercept for probability of known onset date	$\text{Normal}(\mu = 0, \sigma = 2)$	Probability α_1 between 2% and 98%	V
$\sigma_{\text{logit}(\alpha)}$	Standard deviation of random walk on $\text{logit}(\alpha_t)$	$\text{Normal}^+(\mu = 0, \sigma = 0.5)$	Odds for missing onset dates differ between consecutive days maximally by a factor of 3	V
**Observations**				
$\frac{1}{\sqrt{\phi}}$	Overdispersion parameter for case counts	$\text{Normal}^+(\mu = 0, \sigma = 1)$	Generic overdispersion prior for negative binomial [15]	I, II, III, V (optional)
**Exponential smoothing**				
α'	Smoothing parameter for level	$\text{Beta}(\alpha = 5, \beta = 5)$	the last 3–20 days of observations (including the current day) determine over 99% of the current level	I, II, III, V (ETS version)
β'	Smoothing parameter for trend	$\text{Beta}(\alpha = 5, \beta = 5)$	the last 3–20 days of observations (including the current day) determine over 99% of the current trend	I, II, III, V (ETS version)

Normal^+: Normal distribution truncated from below at zero

C Implementation and estimation

C.1 Implementation details

We implemented models I–V as probabilistic programs in Stan [16]. The implementation of model II was adapted from the largely similar model by Günther et al. [3], available at https://github.com/FelixGuenther/nc_covid19_bavaria and improved for efficiency. In particular, we used log scale (or logit scale) versions of the parameters λ_t, $p_{t,d}$, and α_t and propagated them until the final likelihood to reduce computations and to improve numerical stability. We used non-centered parameterizations for the noise components of the random walk and innovations state space models.

For the stepwise approaches, posterior samples were extracted from the fitted model for the previous step and used as data input for the subsequent step. In the case of imputation, we used only a single random sample of the posterior distribution of imputed symptom onset dates from model IV to compute the imputed case counts $\hat{N}_{t,d}$, which were then used as input for model II. In the case of reproduction number estimation, we extracted 50 time series of $\hat{N}_t$ drawn from the posterior predictive distribution of symptom onsets from model II and fitted model I (or EpiEstim) separately on each time series. The resulting samples of the posterior for R_t from the 50 different runs of model I were then combined into an overall posterior distribution of R_t.

In a supplementary analysis, we also estimate R_t with EpiEstim instead of model I. For this, we applied EpiEstim with a centered smoothing window of $\tau = 7$ days separately for each time series draw of $\hat{N}_t$ from the fitted model II, obtaining estimates for the posterior mean and standard deviation of R_t over time. For each draw, we then sampled a trajectory for R_t by drawing from a Gamma distribution with the corresponding mean and standard deviation at each time step. The resulting samples of R_t were then combined into an overall posterior distribution for R_t. Finally, because R_t estimates obtained on the time series of symptom onsets instead of infections lag the actual transmission dynamics [8], we shifted the estimates of R_t back in time by the mean incubation period as well as by half of the smoothing window.

C.2 Sampling

For sampling of Stan models, we used cmdstan version 2.30.0 via cmdstanr version 0.5.0 [17] with the default configuration of the No-U-Turn sampler, i. e. four chains with 1,000 warm-up and 1,000

sampling iterations each [16]. To achieve a faster warm-up, important parameters of the model were initialized using crude empirical estimates from the data. For example, the baseline reporting hazard was initialized using an empirical estimate of the delay distribution ignoring changes over time, weekday effects, and truncation, and the time series of infections was initialized by shifting the time series of observed symptom onsets backward in time by the mean incubation period.

C.3 Diagnostics

Each fitted model was automatically checked for ineffective sampling, indicated by low effective sample sizes (ESS < 400) [18], and for convergence problems, indicated by divergent transitions [19], a low Bayesian fraction of missing information (E-BFMI < 0.2) [20], or high values of the Gelman-Rubin convergence diagnostic ($\hat{R} > 1.01$) [21]. The checks indicated sufficient effective sampling sizes and good convergence and mixing of chains for all fitted models.

D Comparison on synthetic line list data

D.1 Simulation

To obtain a synthetic line list from a single simulation run, we first simulated a stochastic trajectory of infections according to a specified transmission scenario (first or second wave), and then sampled cases with date of symptom onset and reporting delay that are recorded in the line list. Finally, to simulate incomplete line list data, we randomly marked symptom onset dates as missing for some cases. The reporting delays and probabilities of missingness were simulated to change randomly over time and differ across simulation runs. In the following, we provide details for each step of the simulation.

D.1.1 Simulation of infections

Infections were simulated by applying a stochastic renewal model to a given transmission scenario. A transmission scenario in our simulation is characterized by a piecewise linear time series for R_t and a seeding rate of initial infections. The first wave scenario starts with a seeding rate $\mu_{\text{seed}} = 0.5$ and $R_t = 2$. Transmission stays at $R_t = 2$ until day 70, then drops linearly to $R_t = 0.8$ within 10 days, then stays at $R_t = 0.8$ for the remaining time until day 200. The second wave scenario starts with a much higher seeding rate of $\mu_{\text{seed}} = 1000$ and equilibrium-level transmission, i. e. $R_t = 1$. Here the seeding does not necessarily represent imported cases, it is only used to initialize the simulation

of a second wave during ongoing transmission at the epidemic threshold. Transmission stays at $R_t = 1$ until day 70, then linearly increases to $R_t = 1.4$ within 10 days, remains at $R_t = 1.4$ for 20 days, and finally decreases to $R_t = 0.7$ within 30 days and remains at $R_t = 0.7$ for the remaining time until day 200.

For all simulations, we used a generation interval distribution w that follows a discretized Gamma distribution with shape 1.43 and rate 0.29 (i. e. mean 4.9 and standard deviation 4.1 days) [22] and is truncated at a maximum generation time of 21 days. To obtain a simulated trajectory of infections, we sampled initial infections during the first 3 weeks as independently Poisson distributed with rate μ_{seed} on each day. Then, we recursively applied the renewal equation

$$\iota_t = E[I_t] = R_t \sum_{s=1}^{t} \psi_s \, I_{t-s},$$

while sampling realized infections $I_t|\iota_t \sim \text{Poisson}(\iota_t)$ at each time step, which can be interpreted as realizing an age-dependent branching process [11]. The final trajectory of infections was then used to simulate a synthetic line list.

D.1.2 Simulation of line list

Not all infections become reported cases. To mimic the generation of hospitalization line list data, we sampled infections only with a probability of 2% as cases in the line list. For all simulations, we assumed the incubation period to be independent of the generation interval and Gamma distributed with a mean of 5.3 days and a standard deviation of 3.2 days [23]. We drew a random incubation period for each case to simulate its date of symptom onset.

To obtain the corresponding date of report, we drew a reporting delay from a discrete delay distribution. As the baseline delay, we used a discretized lognormal distribution with a mean of 9 days and a standard deviation of 8 days. To simulate differences in the delay distribution over time, we applied the same discrete time-to-event model as used in the nowcasting approaches. Specifically, we determined γ_d, the logit baseline hazard for reporting with d days delay from the above assumed baseline delay distribution. Then, for symptom onset on date t and reporting delay d, we added a proportional-hazards trend effect $\tilde{z}_t$ and a reporting weekday effect $\tilde{w}_{t+d}$ to the logit baseline hazard γ_d, such that the overall hazard is $\text{logit}(h_{t,d}) = \gamma_d + \tilde{z}_t + \tilde{w}_{t+d}$. The trend effect $\tilde{z}_t$ was simulated as a random piecewise linear function with changepoints every 4 weeks, where the values at the

changepoints are drawn from a geometric random walk constrained between ± 0.3, corresponding to a roughly 30% increase or decrease in the hazard odds of reporting. The reporting weekday effect $\tilde{w}_{t+d}$ was chosen such that the hazard odds of reporting are 70% lower on Saturdays, 80% lower on Sundays, 10% higher on Mondays and 5% higher on Tuesdays.

To simulate incomplete line list data with missing symptom onset dates, we selected cases with symptom onset on date t to have a missing onset date with probability $1-\tilde{\alpha}_t$, and let $1-\tilde{\alpha}_t$ vary according to a random piecewise linear function with changepoints every 4 weeks, where the values at the changepoints were drawn from a geometric random walk constrained between 20% and 60% missingness.

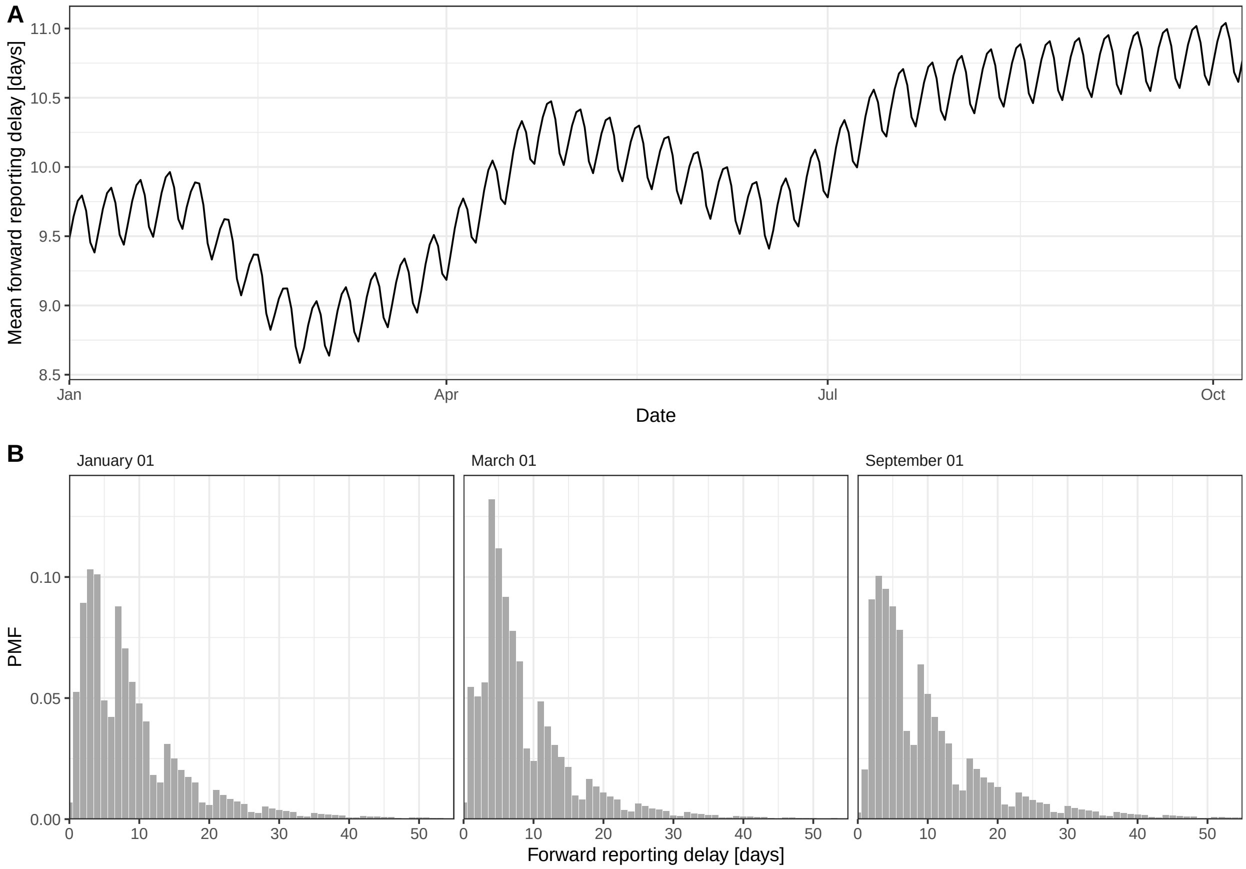

Fig A. Example of a simulated, time-varying delay distribution. (A) Simulated mean forward reporting delay over time, including both weekly seasonality and a linear time trend with changepoints every 4 weeks. (B) Simulated reporting delay distributions shown at selected points in time. The smaller hazard for reporting on weekends shows as a pattern in the forward delay distribution.

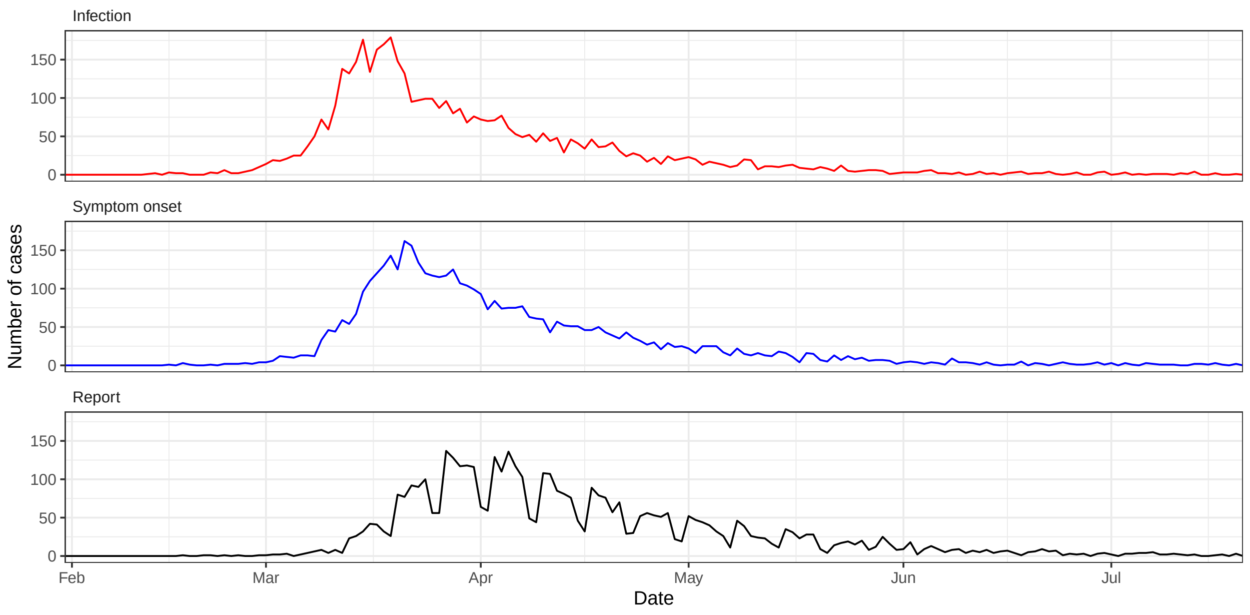

Fig B. Example of a simulated trajectory for the first wave scenario. Shown is the number of eventually reported cases by date of infection (red), date of symptom onset (blue), and date of report (black). The first wave scenario starts with a small number of infections and exponential growth, and then transitions into exponential decline after 70 days. Differences in reporting delay, e. g. reduced reporting on weekends, only affect the time series of cases by date of report.

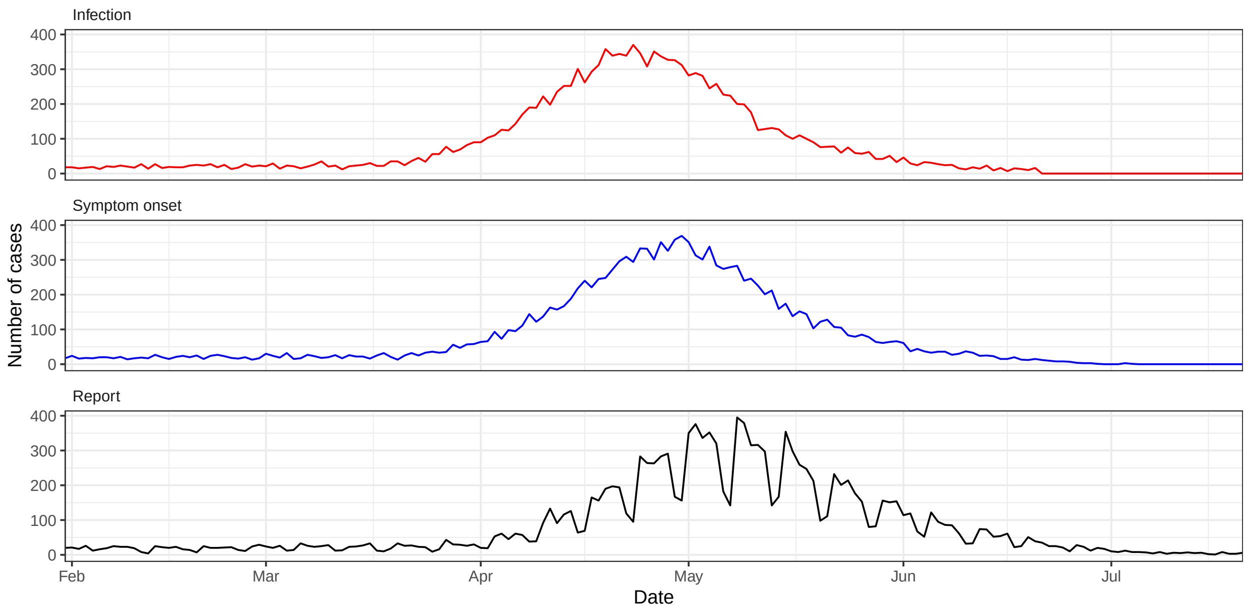

Fig C. Example of a simulated trajectory for the second wave scenario. Shown is the number of eventually reported cases by date of infection (red), date of symptom onset (blue), and date of report (black). The second wave scenario starts with a higher number of infections and equilibrium-level transmission, which increases to exponential growth after 70 days, and finally transitions into exponential decline. Differences in reporting delay, e. g. reduced reporting on weekends, only affect the time series of cases by date of report.

D.2 Performance evaluation

We produced 50 different simulation runs for the first wave and second wave scenario, respectively. For each scenario, we then used the different nowcasting approaches on each of the 50 synthetic line lists to obtain nowcasts for different evaluation time periods. These week-long time periods were chosen to cover different phases of the epidemic wave in each scenario. For the first wave scenario, the evaluation time periods were days 64–70 (before peak I), 77–83 (at peak I), 98–104 (after peak I), and 129–135 (suppression). For the second wave scenario, the evaluation time periods were days 64–70 (control), 94–100 (before peak II), 118–124 (at peak II), 142–148 (After peak II). For each of these 7-day periods, we applied the different nowcasting approaches using data observed until the end of the same week (0–6 days lag), one week later (7–13 days lag), and two weeks later (14–20 days lag).

For nowcasting, we used the generation interval and incubation period distributions assumed in the simulation, i. e. we assumed these parameters to be correctly known. For the comparison of the different approaches of estimating R_t, we used complete synthetic line list data, i.e. no symptom onsets were set to missing. For the comparison of the different approaches of accounting for missing onset dates, we used incomplete synthetic line list data, i.e. missing symptom onsets were simulated as described in Supplement D.1.2.

For each scenario, the above procedure yielded three differently lagged nowcasts for each approach, for each epidemic phase, and for each of the 50 simulation runs. To evaluate the nowcasting performance of the different approaches, we compared each nowcast with the simulated ground truth from the respective synthetic line list. We assessed performance with regard to nowcasting the number of symptom onsets N_t and nowcasting the effective reproduction number R_t, using the weighted interval score (WIS) as described in the main text.

D.3 Supplementary results on synthetic data

In the following, we provide supplementary figures and tables for the results on synthetic data. These include the results of the main analyses for the second wave scenario (Figs D – G in S1 Appendix), results for models with exponential smoothing priors (Figs H – K in S1 Appendix), and results for the stepwise approach using EpiEstim (Figs L and M in S1 Appendix).

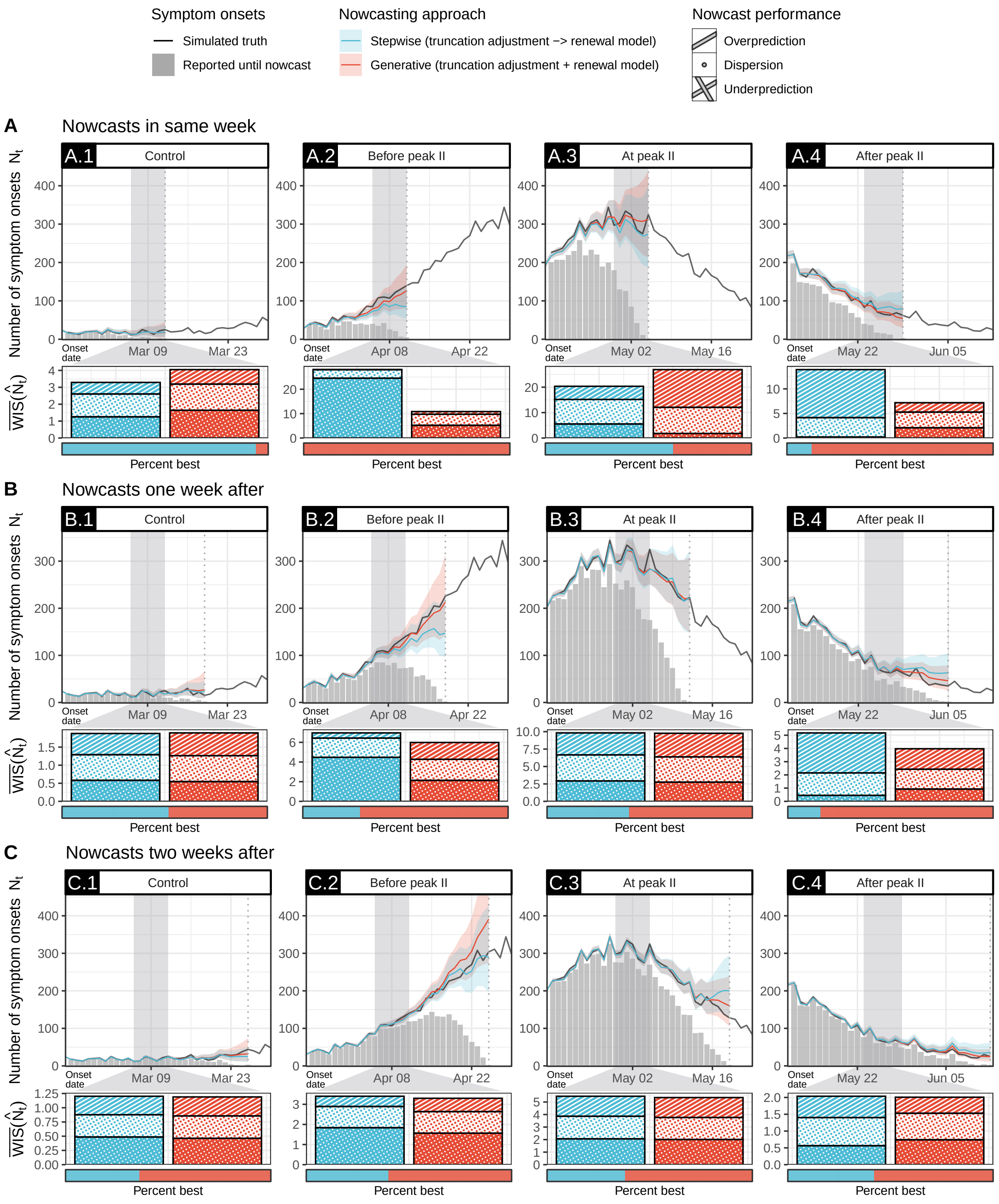

Fig D. Nowcasts of N_t on line list data of a simulated second wave scenario using different approaches of adjusting for right truncation. Shown are the true number of cases by symptom onset date N_t (black), the number of cases reported until the nowcast date (grey bars), and point nowcasts with 95% credible intervals (CrI) in four different phases of the epidemic wave, obtained through i) a stepwise approach using cases by date of symptom onset with a truncation adjustment step (blue), and ii) a generative approach using cases by date of symptom onset with an integrated truncation and renewal model (red). The direct approach using cases by date of report cannot produce nowcasts of N_t. Shown below each phase is the weighted interval score (WIS, lower is better) for N_t nowcasts of each approach during a selected week (grey shade) over 50 scenario runs (see Table B in S1 Appendix for exact figures). Colored bars show average scores, decomposed into penalties for underprediction (crosshatch), dispersion (circles), and overprediction (stripes). dispersion (circles), and overprediction (stripes). The horizontal bar below shows the percentage of times each approach achieved the lowest WIS out of 50 scenario runs, respectively. Results are shown for nowcasts made at different lags from the selected week (vertical dotted lines), i. e. at the end of the selected week (top row), one week later (middle row), and two weeks later (bottom row).

Phase	Lag [days]	Stepwise		Generative	
		$\overline{\text{WIS}}(\hat{N}_t)$	$\%^{\text{best}}$	$\overline{\text{WIS}}(\hat{N}_t)$	$\%^{\text{best}}$
Control	0-6	3.29	94%	4.05	6%
	7-13	1.88	52%	1.89	48%
	14-20	1.20	36%	1.19	64%
Before peak II	0-6	28.05	0%	10.84	100%
	7-13	6.96	28%	5.98	72%
	14-20	3.40	40%	3.30	60%
At peak II	0-6	20.34	62%	26.91	38%
	7-13	9.82	40%	9.76	60%
	14-20	5.46	38%	5.36	62%
After peak II	0-6	13.92	12%	7.18	88%
	7-13	5.17	16%	3.98	84%
	14-20	2.04	42%	2.01	58%

Table B. Performance of nowcasts of N_t on line list data of a simulated second wave scenario using different approaches of adjusting for right truncation. Shown is the performance of N_t nowcasts in four different phases of the epidemic wave, at different lags of a selected week (same week [0–6 days], one week after [7–13 days], and two weeks after [14–20 days]). Nowcasts were obtained through i) a stepwise approach using cases by date of symptom onset with a truncation adjustment step, and ii) a generative approach using cases by date of symptom onset with an integrated truncation and renewal model. Performance is measured by the weighted interval score (WIS, lower is better), shown is the average score over 50 scenario runs ($\overline{\text{WIS}}$) and the percentage of runs in which each approach achieved the best score ($\%^{\text{best}}$).

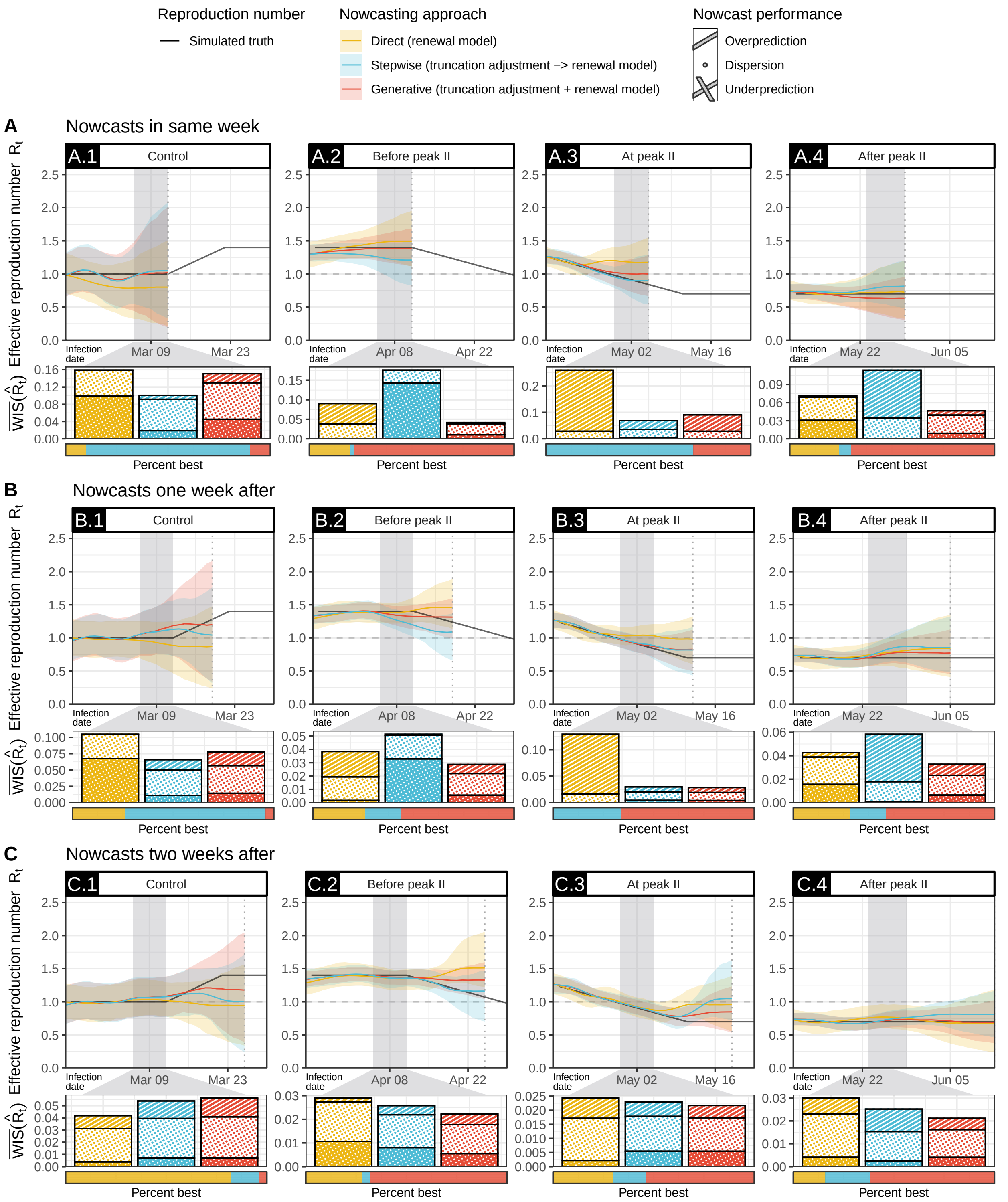

Fig E. Nowcasts of R_t on line list data of a simulated second wave scenario using different approaches of adjusting for right truncation. Shown are the true R_t (black) and point nowcasts with 95% credible intervals (CrI) in four different phases of the epidemic wave, obtained through i) a direct approach using cases by date of report with no truncation adjustment (yellow), ii) a stepwise approach using cases by date of symptom onset with a truncation adjustment step (blue), and iii) a generative approach using cases by date of symptom onset with an integrated truncation and renewal model (red). Shown below each phase is the weighted interval score (WIS, lower is better) for R_t nowcasts of each approach during a selected week (grey shade) over 50 scenario runs (see Table C in S1 Appendix for exact figures). Colored bars show average scores, decomposed into penalties for underprediction (crosshatch), dispersion (circles), and overprediction (stripes). dispersion (circles), and overprediction (stripes). The horizontal bar below shows the percentage of times each approach achieved the lowest WIS out of 50 scenario runs, respectively. Results are shown for nowcasts made at different lags from the selected week (vertical dotted lines), i. e. at the end of the selected week (top row), one week later (middle row), and two weeks later (bottom row).

Phase	Lag [days]	Direct		Stepwise		Generative	
		$\overline{\text{WIS}}(R_t)$	$\%^{\text{best}}$	$\overline{\text{WIS}}(R_t)$	$\%^{\text{best}}$	$\overline{\text{WIS}}(R_t)$	$\%^{\text{best}}$
Control	0-6	0.16	10%	0.10	80%	0.15	10%
	7-13	0.10	26%	0.07	70%	0.08	4%
	14-20	0.04	82%	0.05	14%	0.06	4%
Before peak II	0-6	0.09	20%	0.18	2%	0.04	78%
	7-13	0.04	26%	0.05	18%	0.03	56%
	14-20	0.03	28%	0.03	4%	0.02	68%
At peak II	0-6	0.26	0%	0.07	72%	0.09	28%
	7-13	0.13	0%	0.03	34%	0.03	66%
	14-20	0.02	30%	0.02	16%	0.02	54%
After peak II	0-6	0.07	24%	0.11	6%	0.05	70%
	7-13	0.04	28%	0.06	18%	0.03	54%
	14-20	0.03	16%	0.03	22%	0.02	62%

Table C. Performance of nowcasts of R_t on line list data of a simulated second wave scenario using different approaches of adjusting for right truncation. Shown is the performance of R_t nowcasts in four different phases of the epidemic wave, at different lags of a selected week (same week [0–6 days], one week after [7–13 days], and two weeks after [14–20 days]). Nowcasts were obtained through i) a direct approach using cases by date of report with no truncation adjustment, ii) a stepwise approach using cases by date of symptom onset with a truncation adjustment step, and iii) a generative approach using cases by date of symptom onset with an integrated truncation and renewal model. Performance is measured by the weighted interval score (WIS, lower is better), shown is the average score over 50 scenario runs ($\overline{\text{WIS}}$) and the percentage of runs in which each approach achieved the best score ($\%^{\text{best}}$).

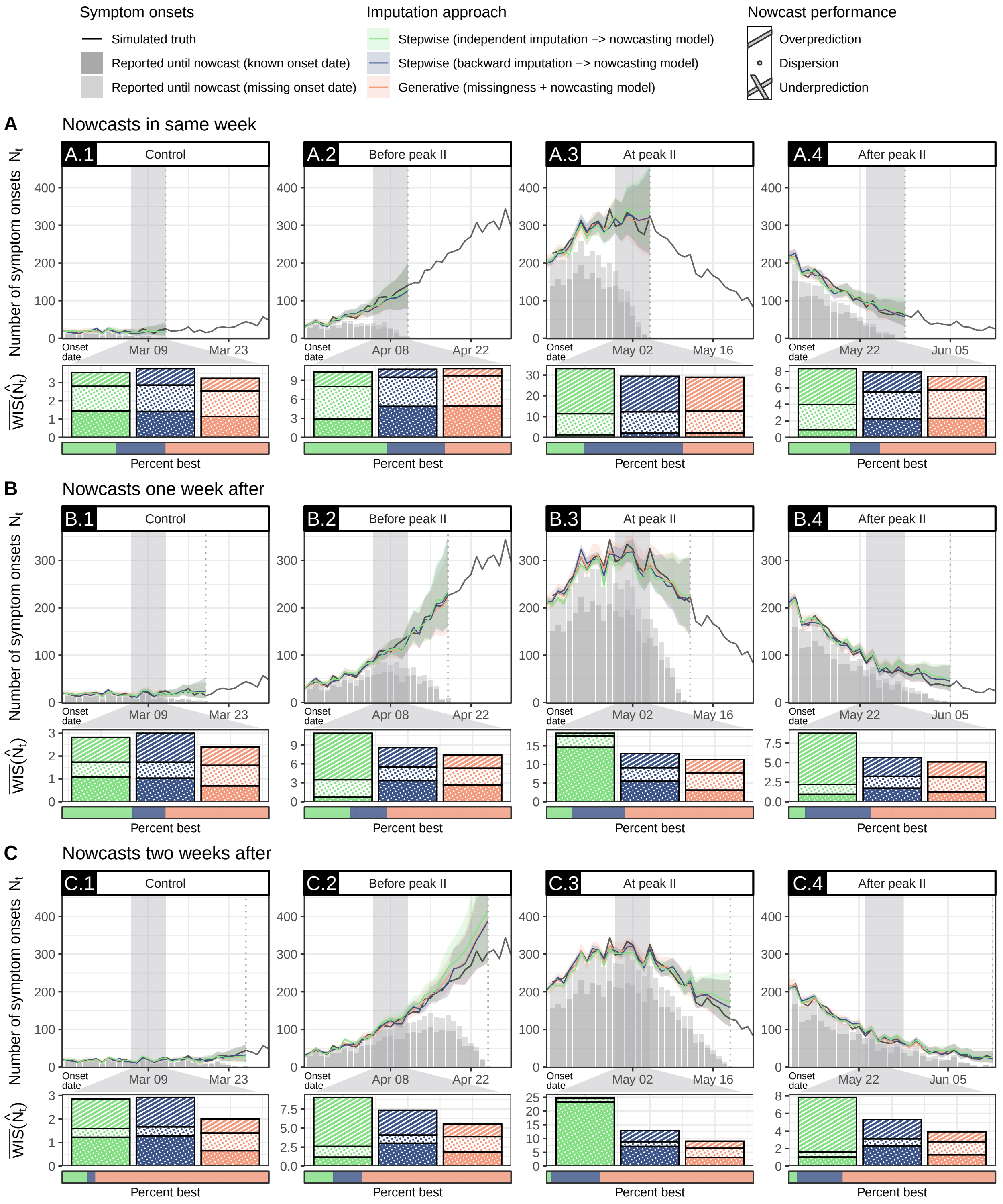

Fig F. Nowcasts of N_t on incomplete line list data of a simulated second wave scenario using different approaches of accounting for missing onset dates. Shown are the true number of cases by symptom onset date N_t (black), the number of cases reported until the nowcast date (dark grey bars for known, light grey bars for missing onset dates), and point nowcasts with 95% credible intervals (CrI) in four different phases of the epidemic wave, obtained through i) a stepwise approach using an independent imputation step (green), ii) a stepwise approach using a backward imputation step (blue), and iii) a generative approach using an integrated missingness model (red). All approaches used a generative model for nowcasting. Shown below each phase is the weighted interval score (WIS, lower is better) for N_t nowcasts of each approach during a selected week (grey shade) over 50 scenario runs (see Table D in S1 Appendix for exact figures). Colored bars show average scores, decomposed into penalties for underprediction (crosshatch), dispersion (circles), and overprediction (stripes). dispersion (circles), and overprediction (stripes). The horizontal bar below shows the percentage of times each approach achieved the lowest WIS out of 50 scenario runs, respectively. Results are shown for nowcasts made at different lags from the selected week (vertical dotted lines), i. e. at the end of the selected week (top row), one week later (middle row), and two weeks later (bottom row).

Phase	Lag [days]	Stepwise (independent)		Stepwise (backward)		Generative	
		$\overline{\text{WIS}}(\hat{N}_t)$	$\%^{\text{best}}$	$\overline{\text{WIS}}(\hat{N}_t)$	$\%^{\text{best}}$	$\overline{\text{WIS}}(\hat{N}_t)$	$\%^{\text{best}}$
Control	0-6	3.56	26%	3.77	24%	3.24	50%
	7-13	2.81	34%	3.00	16%	2.40	50%
	14-20	2.85	12%	2.91	4%	2.00	84%
Before peak II	0-6	10.31	40%	10.78	28%	10.84	32%
	7-13	10.87	22%	8.57	18%	7.40	60%
	14-20	9.01	14%	7.33	14%	5.53	72%
At peak II	0-6	33.04	18%	29.37	48%	28.95	34%
	7-13	18.40	12%	12.90	26%	11.32	62%
	14-20	24.93	2%	12.96	24%	9.08	74%
After peak II	0-6	8.32	30%	7.95	14%	7.36	56%
	7-13	8.75	8%	5.63	32%	5.08	60%
	14-20	7.82	4%	5.29	22%	3.92	74%

Table D. Performance of nowcasts of N_t on incomplete line list data of a simulated second wave scenario using different approaches to account for missing onset dates. Shown is the performance of N_t nowcasts in four different phases of the epidemic wave, at different lags of a selected week (same week [0–6 days], one week after [7–13 days], and two weeks after [14–20 days]). Nowcasts were obtained through i) a stepwise approach using an independent imputation step, ii) a stepwise approach using a backward imputation step, and iii) a generative approach using an integrated missingness model. Performance is measured by the weighted interval score (WIS, lower is better), shown is the average score over 50 scenario runs ($\overline{\text{WIS}}$) and the percentage of runs in which each approach achieved the best score ($\%^{\text{best}}$).

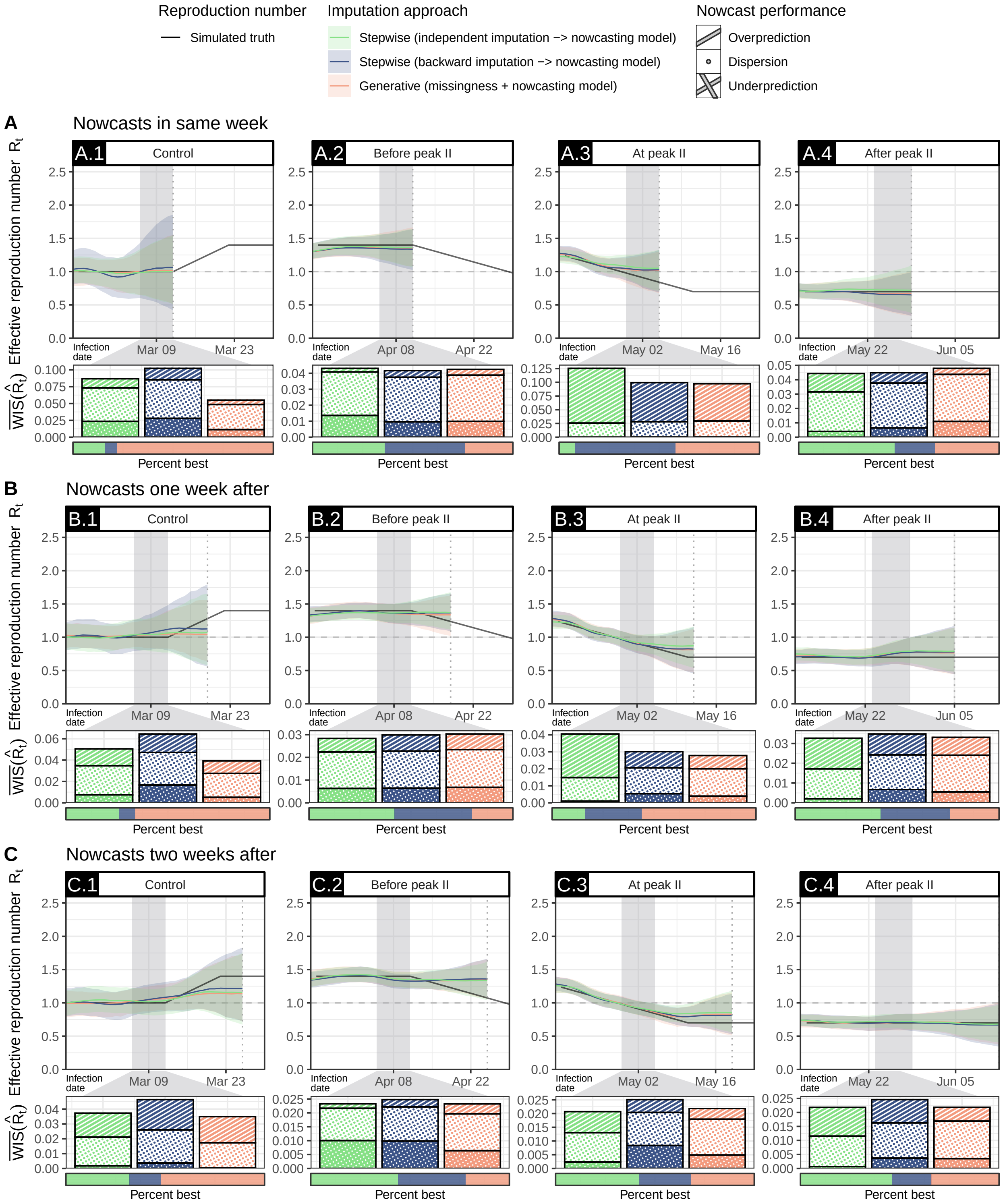

Fig G. Nowcasts of R_t on incomplete line list data of a simulated second wave scenario using different approaches of accounting for missing onset dates. Shown are the true R_t (black) and point nowcasts with 95% credible intervals (CrI) in four different phases of the epidemic wave, obtained through i) a stepwise approach using an independent imputation step (green), ii) a stepwise approach using a backward imputation step (blue), and iii) a generative approach using an integrated missingness model (red). All approaches used a generative model for nowcasting. Shown below each phase is the weighted interval score (WIS, lower is better) for R_t nowcasts of each approach during a selected week (grey shade) over 50 scenario runs (see Table E in S1 Appendix for exact figures). Colored bars show average scores, decomposed into penalties for underprediction (crosshatch), dispersion (circles), and overprediction (stripes). dispersion (circles), and overprediction (stripes). The horizontal bar below shows the percentage of times each approach achieved the lowest WIS out of 50 scenario runs, respectively. Results are shown for nowcasts made at different lags from the selected week (vertical dotted lines), i. e. at the end of the selected week (top row), one week later (middle row), and two weeks later (bottom row).

Phase	Lag [days]	Stepwise (independent)		Stepwise (backward)		Generative	
		$\overline{\text{WIS}}(R_t)$	$\%^{\text{best}}$	$\overline{\text{WIS}}(R_t)$	$\%^{\text{best}}$	$\overline{\text{WIS}}(R_t)$	$\%^{\text{best}}$
Control	0-6	0.09	16%	0.10	6%	0.06	78%
	7-13	0.05	26%	0.06	8%	0.04	66%
	14-20	0.04	32%	0.05	16%	0.04	52%
Before peak II	0-6	0.04	36%	0.04	40%	0.04	24%
	7-13	0.03	42%	0.03	38%	0.03	20%
	14-20	0.02	44%	0.03	34%	0.02	22%
At peak II	0-6	0.12	8%	0.10	50%	0.10	42%
	7-13	0.04	16%	0.03	28%	0.03	56%
	14-20	0.02	42%	0.03	26%	0.02	32%
After peak II	0-6	0.04	48%	0.04	20%	0.05	32%
	7-13	0.03	42%	0.04	34%	0.03	24%
	14-20	0.02	46%	0.03	20%	0.02	34%

Table E. Performance of nowcasts of R_t on incomplete line list data of a simulated second wave scenario using different approaches to account for missing onset dates. Shown is the performance of R_t nowcasts in four different phases of the epidemic wave, at different lags of a selected week (same week [0–6 days], one week after [7–13 days], and two weeks after [14–20 days]). Nowcasts were obtained through i) a stepwise approach using an independent imputation step, ii) a stepwise approach using a backward imputation step, and iii) a generative approach using an integrated missingness model. Performance is measured by the weighted interval score (WIS, lower is better), shown is the average score over 50 scenario runs ($\overline{\text{WIS}}$) and the percentage of runs in which each approach achieved the best score ($\%^{\text{best}}$).

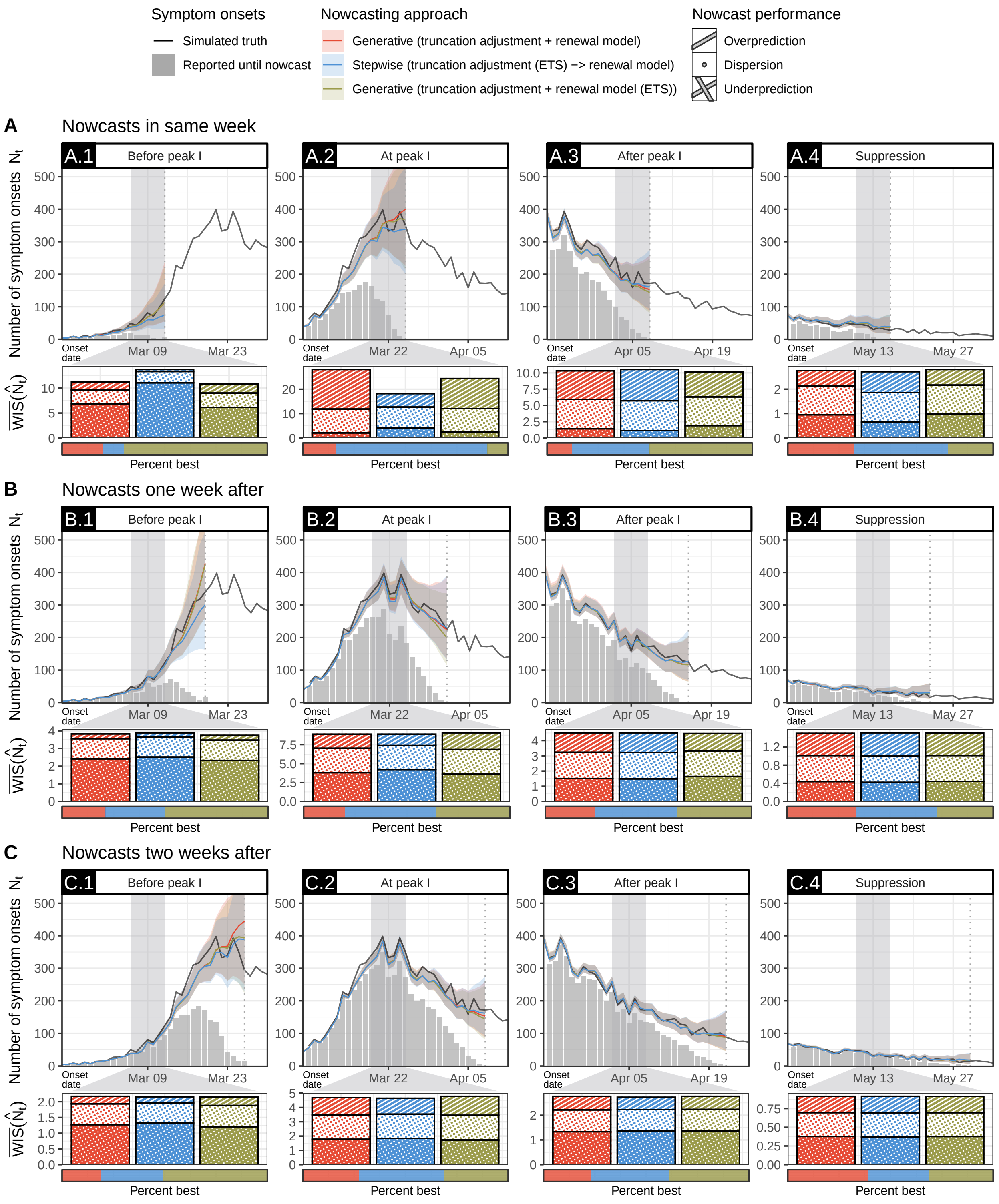

Fig H. Nowcasts of N_t on line list data of a simulated first wave scenario using different approaches of adjusting for right truncation with stationary and non-stationary smoothing priors. Shown are the true number of cases by symptom onset date N_t (black), the number of cases reported until the nowcast date (grey bars), and point nowcasts with 95% credible intervals (CrI) in four different phases of the epidemic wave, obtained through i) a generative approach with a stationary random walk prior on R_t (red, same as main text) ii) a stepwise approach with a non-stationary exponential smoothing prior on λ_t (blue), and iii) a generative approach with a non-stationary exponential smoothing prior on R_t (beige). Shown below each phase is the weighted interval score (WIS, lower is better) for N_t nowcasts of each approach during a selected week (grey shade) over 50 scenario runs (see Table F in S1 Appendix for exact figures). Colored bars show average scores, decomposed into penalties for underprediction (crosshatch), dispersion (circles), and overprediction (stripes). dispersion (circles), and overprediction (stripes). The horizontal bar below shows the percentage of times each approach achieved the lowest WIS out of 50 scenario runs, respectively. Results are shown for nowcasts made at different lags from the selected week (vertical dotted lines), i. e. at the end of the selected week (top row), one week later (middle row), and two weeks later (bottom row).

Phase	Lag [days]	Generative		Stepwise (non-stationary)		Generative (non-stationary)	
		$\overline{\text{WIS}}(\hat{N}_t)$	$\%^{\text{best}}$	$\overline{\text{WIS}}(\hat{N}_t)$	$\%^{\text{best}}$	$\overline{\text{WIS}}(\hat{N}_t)$	$\%^{\text{best}}$
Before peak I	0-6	11.23	20%	13.73	10%	10.80	70%
	7-13	3.82	21%	3.89	29%	3.75	50%
	14-20	2.17	19%	2.15	30%	2.15	51%
At peak I	0-6	28.10	16%	18.18	74%	24.42	10%
	7-13	8.88	20%	8.89	44%	9.05	36%
	14-20	4.69	27%	4.64	41%	4.78	31%
After peak I	0-6	10.31	12%	10.51	38%	10.12	50%
	7-13	4.51	24%	4.51	40%	4.47	36%
	14-20	2.77	23%	2.73	38%	2.77	39%
Suppression	0-6	2.76	32%	2.71	46%	2.80	22%
	7-13	1.50	33%	1.51	39%	1.51	27%
	14-20	0.91	39%	0.91	30%	0.91	31%

Table F. Performance of nowcasts of N_t on line list data of a simulated first wave scenario using different approaches of adjusting for right truncation with stationary and non-stationary smoothing priors. Shown is the performance of N_t nowcasts in four different phases of the epidemic wave, at different lags of a selected week (same week [0–6 days], one week after [7–13 days], and two weeks after [14–20 days]). Nowcasts were obtained through i) a generative approach with a stationary random walk prior on R_t (same as main text) ii) a stepwise approach with a non-stationary exponential smoothing prior on λ_t, and iii) a generative approach with a non-stationary exponential smoothing prior on R_t. Performance is measured by the weighted interval score (WIS, lower is better), shown is the average score over 50 scenario runs ($\overline{\text{WIS}}$) and the percentage of runs in which each approach achieved the best score ($\%^{\text{best}}$).

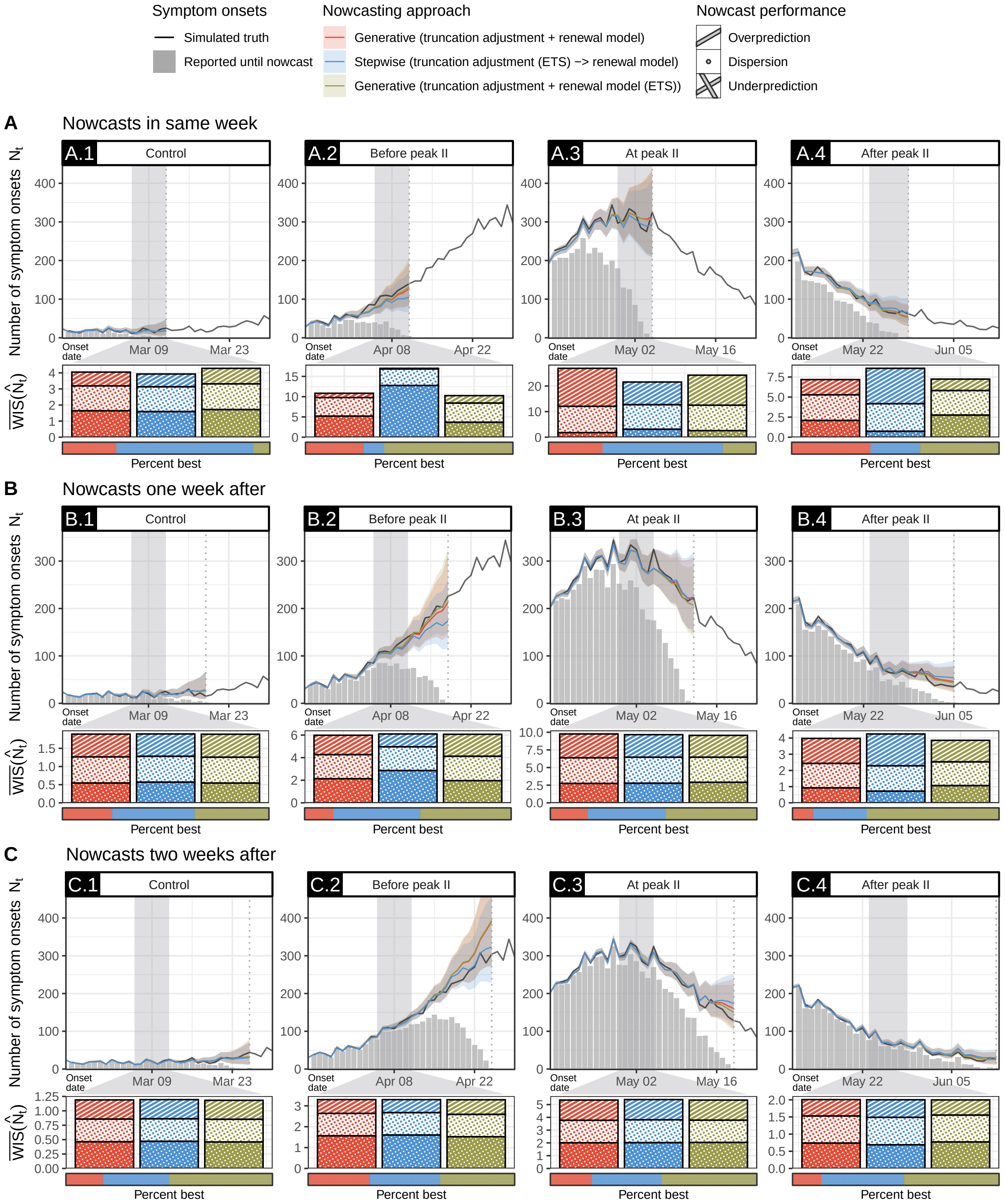

Fig I. Nowcasts of N_t on line list data of a simulated second wave scenario using different approaches of adjusting for right truncation with stationary and non-stationary smoothing priors. Shown are the true number of cases by symptom onset date N_t (black), the number of cases reported until the nowcast date (grey bars), and point nowcasts with 95% credible intervals (CrI) in four different phases of the epidemic wave, obtained through i) a generative approach with a stationary random walk prior on R_t (red, same as main text) ii) a stepwise approach with a non-stationary exponential smoothing prior on λ_t (blue), and iii) a generative approach with a non-stationary exponential smoothing prior on R_t (beige). Shown below each phase is the weighted interval score (WIS, lower is better) for N_t nowcasts of each approach during a selected week (grey shade) over 50 scenario runs (see Table G in S1 Appendix for exact figures). Colored bars show average scores, decomposed into penalties for underprediction (crosshatch), dispersion (circles), and overprediction (stripes). dispersion (circles), and overprediction (stripes). The horizontal bar below shows the percentage of times each approach achieved the lowest WIS out of 50 scenario runs, respectively. Results are shown for nowcasts made at different lags from the selected week (vertical dotted lines), i.e. at the end of the selected week (top row), one week later (middle row), and two weeks later (bottom row).

Phase	Lag [days]	Generative		Stepwise (non-stationary)		Generative (non-stationary)	
		$\overline{\text{WIS}}(\hat{N}_t)$	$\%^{\text{best}}$	$\overline{\text{WIS}}(\hat{N}_t)$	$\%^{\text{best}}$	$\overline{\text{WIS}}(\hat{N}_t)$	$\%^{\text{best}}$
Control	0-6	4.05	26%	3.93	66%	4.28	8%
	7-13	1.89	24%	1.90	40%	1.89	36%
	14-20	1.19	18%	1.20	32%	1.18	50%
Before peak II	0-6	10.84	28%	17.00	10%	10.26	62%
	7-13	5.98	14%	6.10	42%	6.07	44%
	14-20	3.30	30%	3.30	34%	3.29	36%
At peak II	0-6	26.91	26%	21.55	58%	24.23	16%
	7-13	9.76	18%	9.66	38%	9.56	44%
	14-20	5.36	20%	5.39	34%	5.35	46%
After peak II	0-6	7.18	38%	8.59	24%	7.24	38%
	7-13	3.98	10%	4.25	26%	3.86	64%
	14-20	2.01	14%	2.00	40%	2.00	46%

Table G. Performance of nowcasts of N_t on line list data of a simulated second wave scenario using different approaches of adjusting for right truncation with stationary and non-stationary smoothing priors. Shown is the performance of N_t nowcasts in four different phases of the epidemic wave, at different lags of a selected week (same week [0–6 days], one week after [7–13 days], and two weeks after [14–20 days]). Nowcasts were obtained through i) a generative approach with a stationary random walk prior on R_t (same as main text) ii) a stepwise approach with a non-stationary exponential smoothing prior on λ_t, and iii) a generative approach with a non-stationary exponential smoothing prior on R_t. Performance is measured by the weighted interval score (WIS, lower is better), shown is the average score over 50 scenario runs ($\overline{\text{WIS}}$) and the percentage of runs in which each approach achieved the best score ($\%^{\text{best}}$).

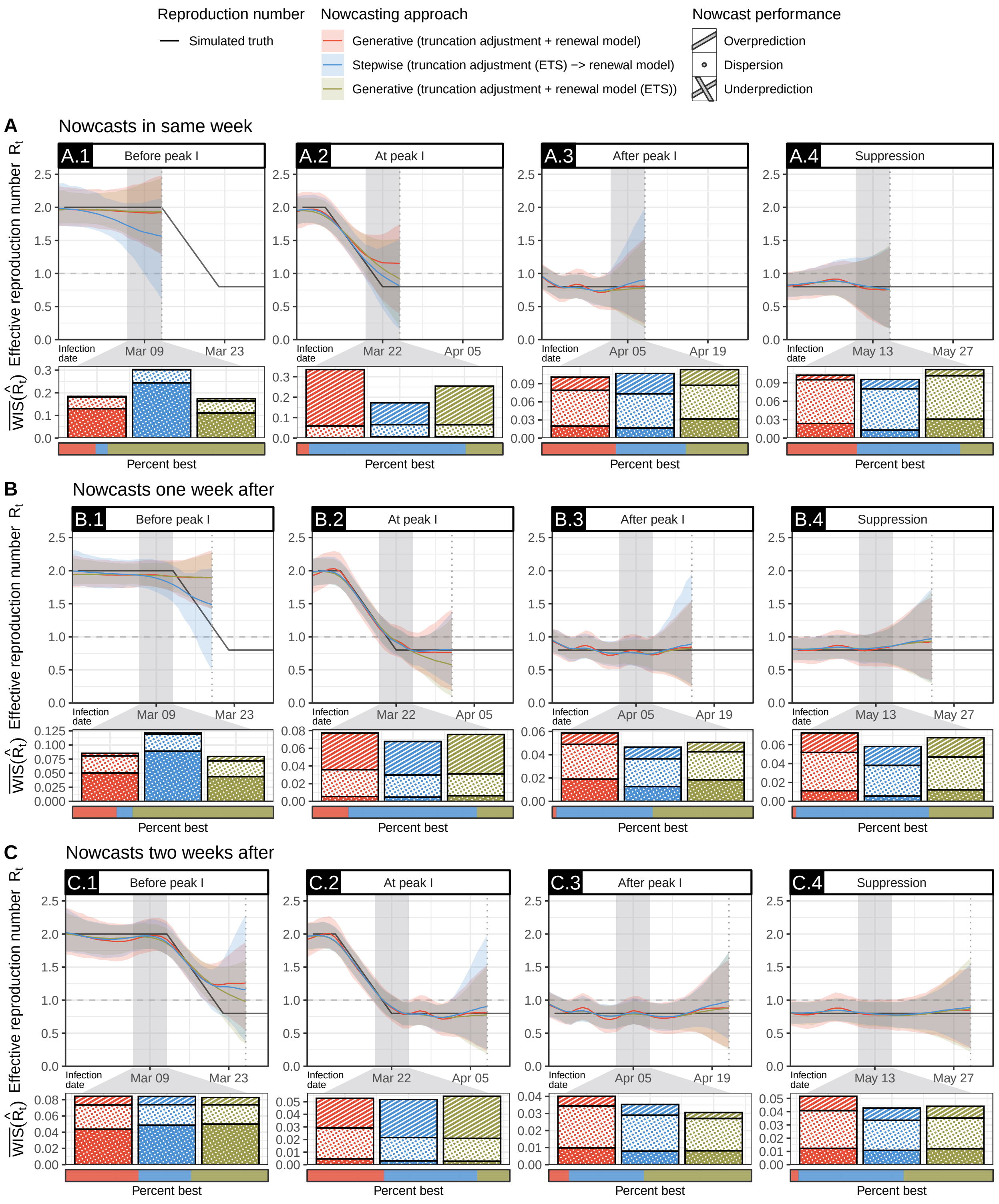

Fig J. Nowcasts of R_t on line list data of a simulated first wave scenario using different approaches of adjusting for right truncation with stationary and non-stationary smoothing priors. Shown are the true R_t (black) and point nowcasts with 95% credible intervals (CrI) in four different phases of the epidemic wave, obtained through i) a generative approach with a stationary random walk prior on R_t (red, same as main text) ii) a stepwise approach with a non-stationary exponential smoothing prior on λ_t (blue), and iii) a generative approach with a non-stationary exponential smoothing prior on R_t (beige). Shown below each phase is the weighted interval score (WIS, lower is better) for R_t nowcasts of each approach during a selected week (grey shade) over 50 scenario runs (see Table H in S1 Appendix for exact figures). Colored bars show average scores, decomposed into penalties for underprediction (crosshatch), dispersion (circles), and overprediction (stripes). dispersion (circles), and overprediction (stripes). The horizontal bar below shows the percentage of times each approach achieved the lowest WIS out of 50 scenario runs, respectively. Results are shown for nowcasts made at different lags from the selected week (vertical dotted lines), i. e. at the end of the selected week (top row), one week later (middle row), and two weeks later (bottom row).

Phase	Lag [days]	Generative		Stepwise (non-stationary)		Generative (non-stationary)	
		$\overline{\text{WIS}}(R_t)$	$\%^{\text{best}}$	$\overline{\text{WIS}}(R_t)$	$\%^{\text{best}}$	$\overline{\text{WIS}}(R_t)$	$\%^{\text{best}}$
Before peak I	0-6	0.18	18%	0.30	6%	0.17	76%
	7-13	0.09	22%	0.12	8%	0.08	70%
	14-20	0.08	36%	0.08	26%	0.08	38%
At peak I	0-6	0.34	6%	0.17	76%	0.25	18%
	7-13	0.08	18%	0.07	64%	0.08	18%
	14-20	0.05	38%	0.05	46%	0.05	16%
After peak I	0-6	0.10	36%	0.11	34%	0.11	30%
	7-13	0.06	2%	0.05	48%	0.05	50%
	14-20	0.04	10%	0.04	37%	0.03	53%
Suppression	0-6	0.10	34%	0.10	50%	0.11	16%
	7-13	0.07	2%	0.06	66%	0.07	32%
	14-20	0.05	4%	0.04	52%	0.04	44%

Table H. Performance of nowcasts of R_t on line list data of a simulated first wave scenario using different approaches of adjusting for right truncation with stationary and non-stationary smoothing priors. Shown is the performance of R_t nowcasts in four different phases of the epidemic wave, at different lags of a selected week (same week [0–6 days], one week after [7–13 days], and two weeks after [14–20 days]). Nowcasts were obtained through i) a generative approach with a stationary random walk prior on R_t (same as main text) ii) a stepwise approach with a non-stationary exponential smoothing prior on λ_t, and iii) a generative approach with a non-stationary exponential smoothing prior on R_t. Performance is measured by the weighted interval score (WIS, lower is better), shown is the average score over 50 scenario runs ($\overline{\text{WIS}}$) and the percentage of runs in which each approach achieved the best score ($\%^{\text{best}}$).

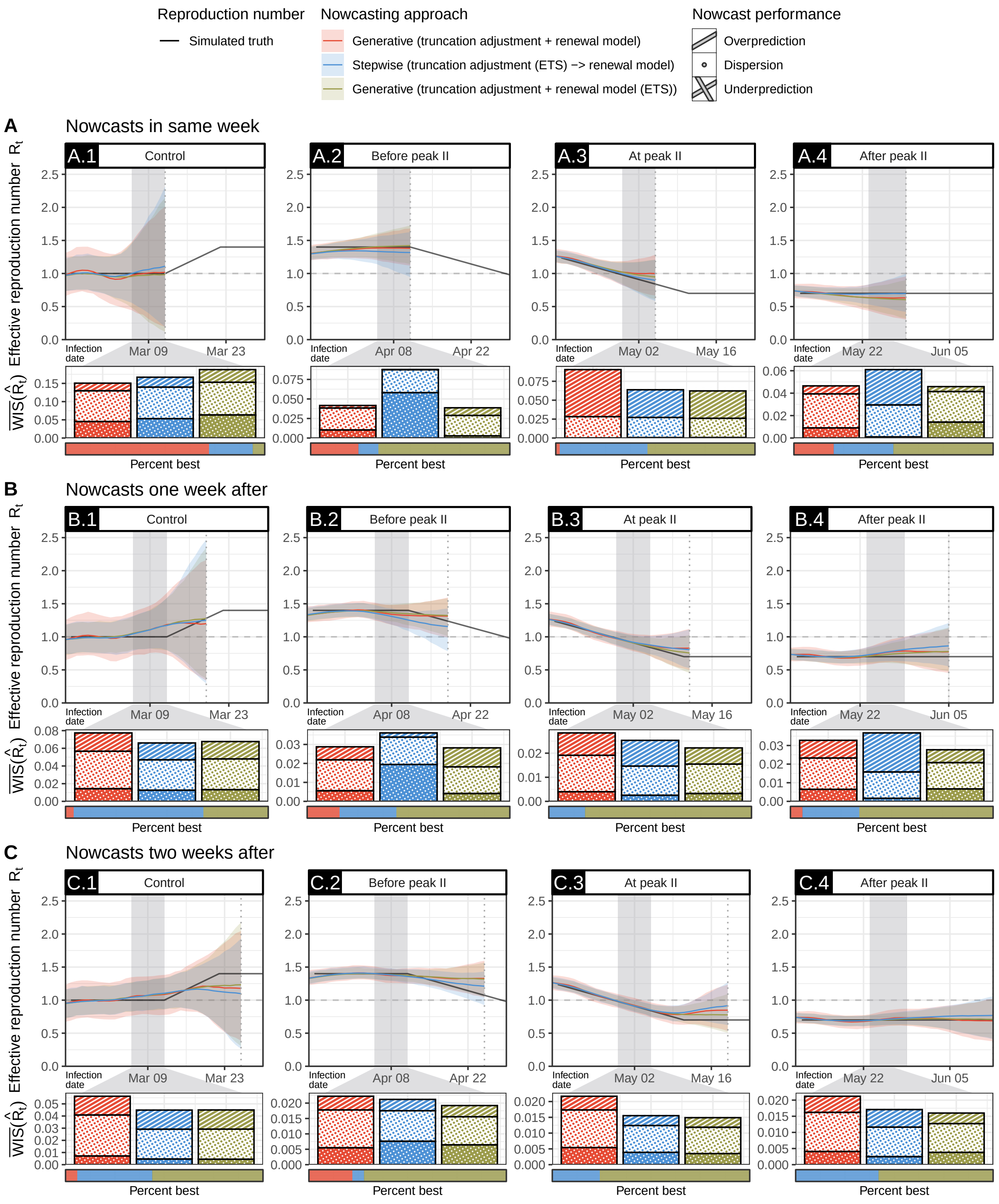

Fig K. Nowcasts of R_t on line list data of a simulated second wave scenario using different approaches of adjusting for right truncation with stationary and non-stationary smoothing priors. Shown are the true R_t (black) and point nowcasts with 95% credible intervals (CrI) in four different phases of the epidemic wave, obtained through i) a generative approach with a stationary random walk prior on R_t (red, same as main text) ii) a stepwise approach with a non-stationary exponential smoothing prior on λ_t (blue), and iii) a generative approach with a non-stationary exponential smoothing prior on R_t (beige). Shown below each phase is the weighted interval score (WIS, lower is better) for R_t nowcasts of each approach during a selected week (grey shade) over 50 scenario runs (see Table I in S1 Appendix for exact figures). Colored bars show average scores, decomposed into penalties for underprediction (crosshatch), dispersion (circles), and overprediction (stripes). dispersion (circles), and overprediction (stripes). The horizontal bar below shows the percentage of times each approach achieved the lowest WIS out of 50 scenario runs, respectively. Results are shown for nowcasts made at different lags from the selected week (vertical dotted lines), i. e. at the end of the selected week (top row), one week later (middle row), and two weeks later (bottom row).

Phase	Lag [days]	Generative		Stepwise (non-stationary)		Generative (non-stationary)	
		$\overline{\text{WIS}}(R_t)$	$\%^{\text{best}}$	$\overline{\text{WIS}}(R_t)$	$\%^{\text{best}}$	$\overline{\text{WIS}}(R_t)$	$\%^{\text{best}}$
Control	0-6	0.15	72%	0.17	22%	0.19	6%
	7-13	0.08	4%	0.07	64%	0.07	32%
	14-20	0.06	6%	0.04	38%	0.04	56%
Before peak II	0-6	0.04	24%	0.09	10%	0.04	66%
	7-13	0.03	16%	0.04	28%	0.03	56%
	14-20	0.02	22%	0.02	6%	0.02	72%
At peak II	0-6	0.09	2%	0.06	44%	0.06	54%
	7-13	0.03	0%	0.03	18%	0.02	82%
	14-20	0.02	0%	0.02	24%	0.01	76%
After peak II	0-6	0.05	20%	0.06	30%	0.05	50%
	7-13	0.03	6%	0.04	28%	0.03	66%
	14-20	0.02	0%	0.02	42%	0.02	58%

Table I. Performance of nowcasts of R_t on line list data of a simulated second wave scenario using different approaches of adjusting for right truncation with stationary and non-stationary smoothing priors. Shown is the performance of R_t nowcasts in four different phases of the epidemic wave, at different lags of a selected week (same week [0–6 days], one week after [7–13 days], and two weeks after [14–20 days]). Nowcasts were obtained through i) a generative approach with a stationary random walk prior on R_t (same as main text) ii) a stepwise approach with a non-stationary exponential smoothing prior on λ_t, and iii) a generative approach with a non-stationary exponential smoothing prior on R_t. Performance is measured by the weighted interval score (WIS, lower is better), shown is the average score over 50 scenario runs ($\overline{\text{WIS}}$) and the percentage of runs in which each approach achieved the best score ($\%^{\text{best}}$).

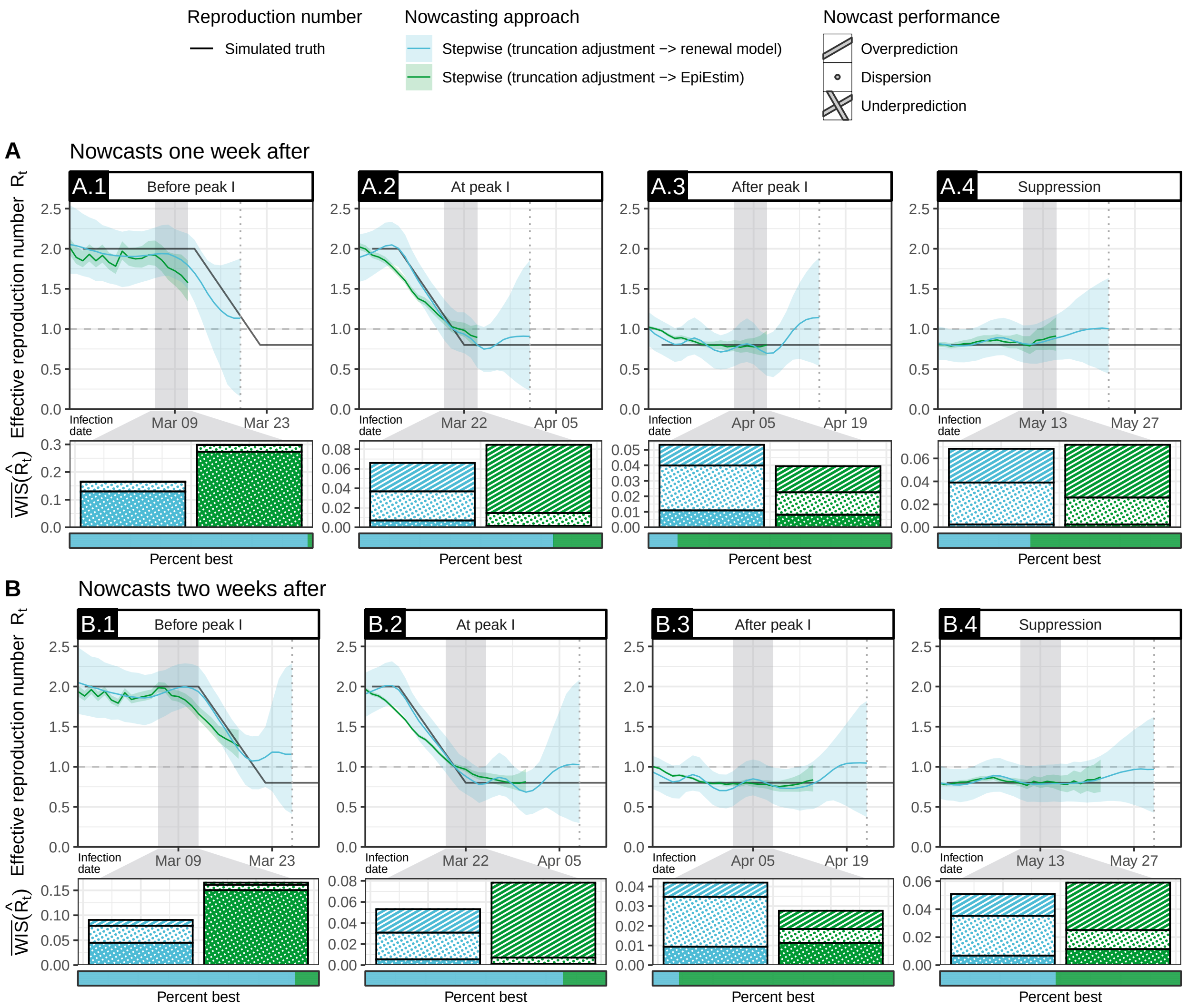

Fig L. Nowcasts of R_t on line list data of a simulated first wave scenario using a stepwise approach with a renewal model and EpiEstim for R_t estimation. Shown are the true R_t (black) and point nowcasts with 95% credible intervals (CrI) in four different phases of the epidemic wave, obtained through i) a stepwise approach using a renewal model for R_t estimation (blue), and ii) a stepwise approach using EpiEstim with a smoothing window of 7 days for R_t estimation (green). Shown below each phase is the weighted interval score (WIS, lower is better) for R_t nowcasts of each approach during a selected week (grey shade) over 50 scenario runs (see Table J in S1 Appendix for exact figures). Colored bars show average scores, decomposed into penalties for underprediction (crosshatch), dispersion (circles), and overprediction (stripes). dispersion (circles), and overprediction (stripes). The horizontal bar below shows the percentage of times each approach achieved the lowest WIS out of 50 scenario runs, respectively. Results are shown for nowcasts made at different lags from the selected week (vertical dotted lines), i. e. one week later (middle row), and two weeks later (bottom row). Nowcasts using EpiEstim were shifted backward in time to account for the incubation period and to center the smoothing window of EpiEstim, and are therefore only available at a lag of 8 days or longer.

Phase	Lag [days]	Stepwise		Stepwise (EpiEstim)	
		$\overline{\text{WIS}}(R_t)$	$\%^{\text{best}}$	$\overline{\text{WIS}}(R_t)$	$\%^{\text{best}}$
Before peak I	8-13	0.17	98%	0.30	2%
	14-20	0.09	90%	0.17	10%
At peak I	8-13	0.07	80%	0.09	20%
	14-20	0.05	82%	0.08	18%
After peak I	8-13	0.05	12%	0.04	88%
	14-20	0.04	11%	0.03	89%
Suppression	8-13	0.07	38%	0.07	62%
	14-20	0.05	48%	0.06	52%

Table J. Performance of nowcasts of R_t on line list data of a simulated first wave scenario using a stepwise approach with a renewal model and EpiEstim for R_t estimation. Shown is the performance of R_t nowcasts in four different phases of the epidemic wave, at different lags of a selected week (same week [0–6 days], one week after [7–13 days], and two weeks after [14–20 days]). Nowcasts were obtained through i) a stepwise approach using a renewal model for R_t estimation, and ii) a stepwise approach using EpiEstim with a smoothing window of 7 days for R_t estimation. Performance is measured by the weighted interval score (WIS, lower is better), shown is the average score over 50 scenario runs ($\overline{\text{WIS}}$) and the percentage of runs in which each approach achieved the best score ($\%^{\text{best}}$).

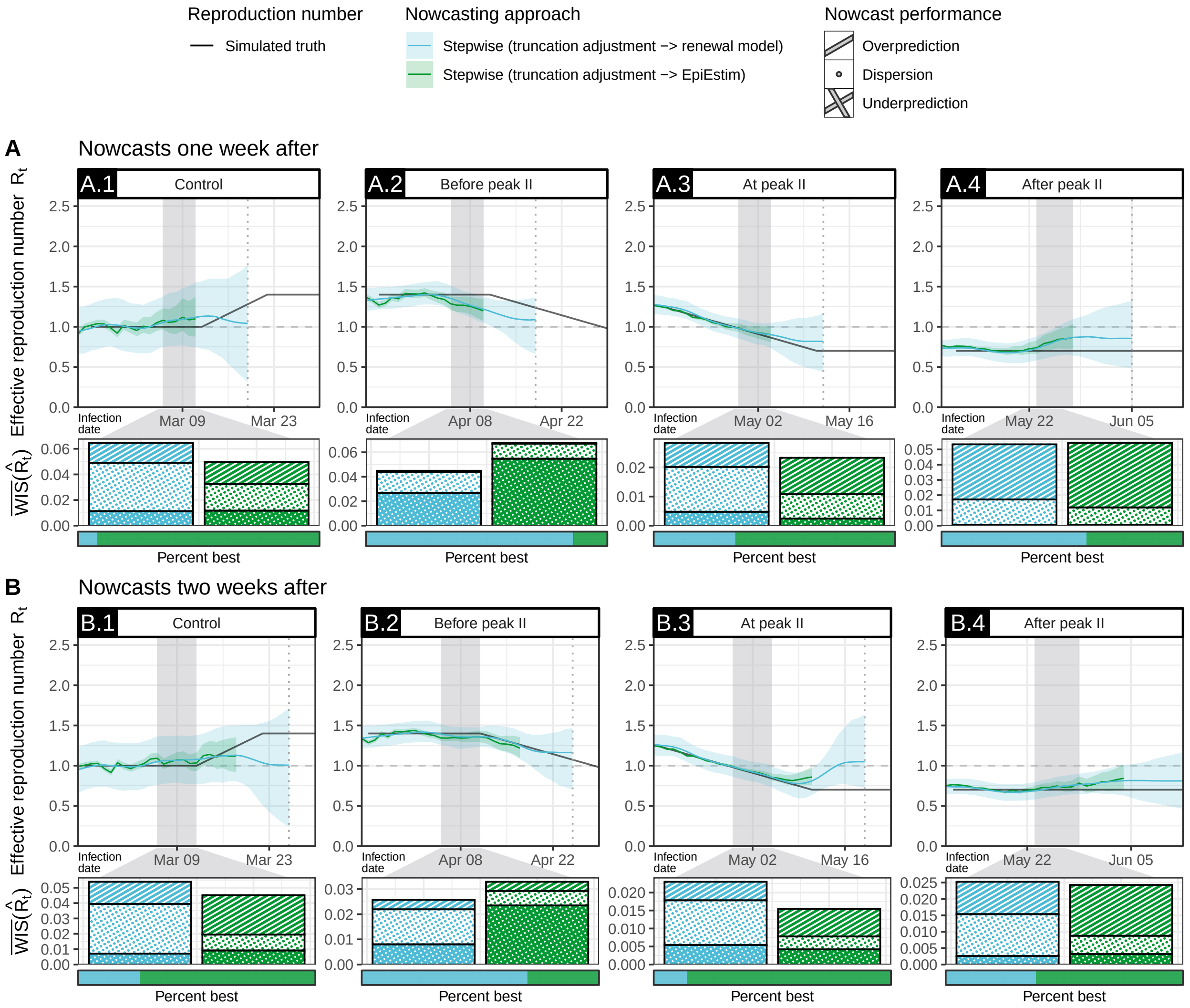

Fig M. Nowcasts of R_t on line list data of a simulated second wave scenario using a stepwise approach with a renewal model and EpiEstim for R_t estimation. Shown are the true R_t (black) and point nowcasts with 95% credible intervals (CrI) in four different phases of the epidemic wave, obtained through i) a stepwise approach using a renewal model for R_t estimation (blue), and ii) a stepwise approach using EpiEstim with a smoothing window of 7 days for R_t estimation (green). Shown below each phase is the weighted interval score (WIS, lower is better) for R_t nowcasts of each approach during a selected week (grey shade) over 50 scenario runs (see Table K in S1 Appendix for exact figures). Colored bars show average scores, decomposed into penalties for underprediction (crosshatch), dispersion (circles), and overprediction (stripes). dispersion (circles), and overprediction (stripes). The horizontal bar below shows the percentage of times each approach achieved the lowest WIS out of 50 scenario runs, respectively. Results are shown for nowcasts made at different lags from the selected week (vertical dotted lines), i. e. at the end of the selected week (top row), one week later (middle row), and two weeks later (bottom row).

Phase	Lag [days]	Stepwise		Stepwise (EpiEstim)	
		$\overline{\text{WIS}}(R_t)$	$\%^{\text{best}}$	$\overline{\text{WIS}}(R_t)$	$\%^{\text{best}}$
Control	8-13	0.07	8%	0.05	92%
	14-20	0.05	26%	0.04	74%
Before peak II	8-13	0.04	86%	0.07	14%
	14-20	0.03	70%	0.03	30%
At peak II	8-13	0.03	34%	0.02	66%
	14-20	0.02	14%	0.01	86%
After peak II	8-13	0.05	60%	0.05	40%
	14-20	0.03	38%	0.02	62%

Table K. Performance of nowcasts of R_t on line list data of a simulated second wave scenario using a stepwise approach with a renewal model and EpiEstim for R_t estimation. Shown is the performance of R_t nowcasts in four different phases of the epidemic wave, at different lags of a selected week (same week [0–6 days], one week after [7–13 days], and two weeks after [14–20 days]). Nowcasts were obtained through i) a stepwise approach using a renewal model for R_t estimation, and ii) a stepwise approach using EpiEstim with a smoothing window of 7 days for R_t estimation. Performance is measured by the weighted interval score (WIS, lower is better), shown is the average score over 50 scenario runs ($\overline{\text{WIS}}$) and the percentage of runs in which each approach achieved the best score ($\%^{\text{best}}$).

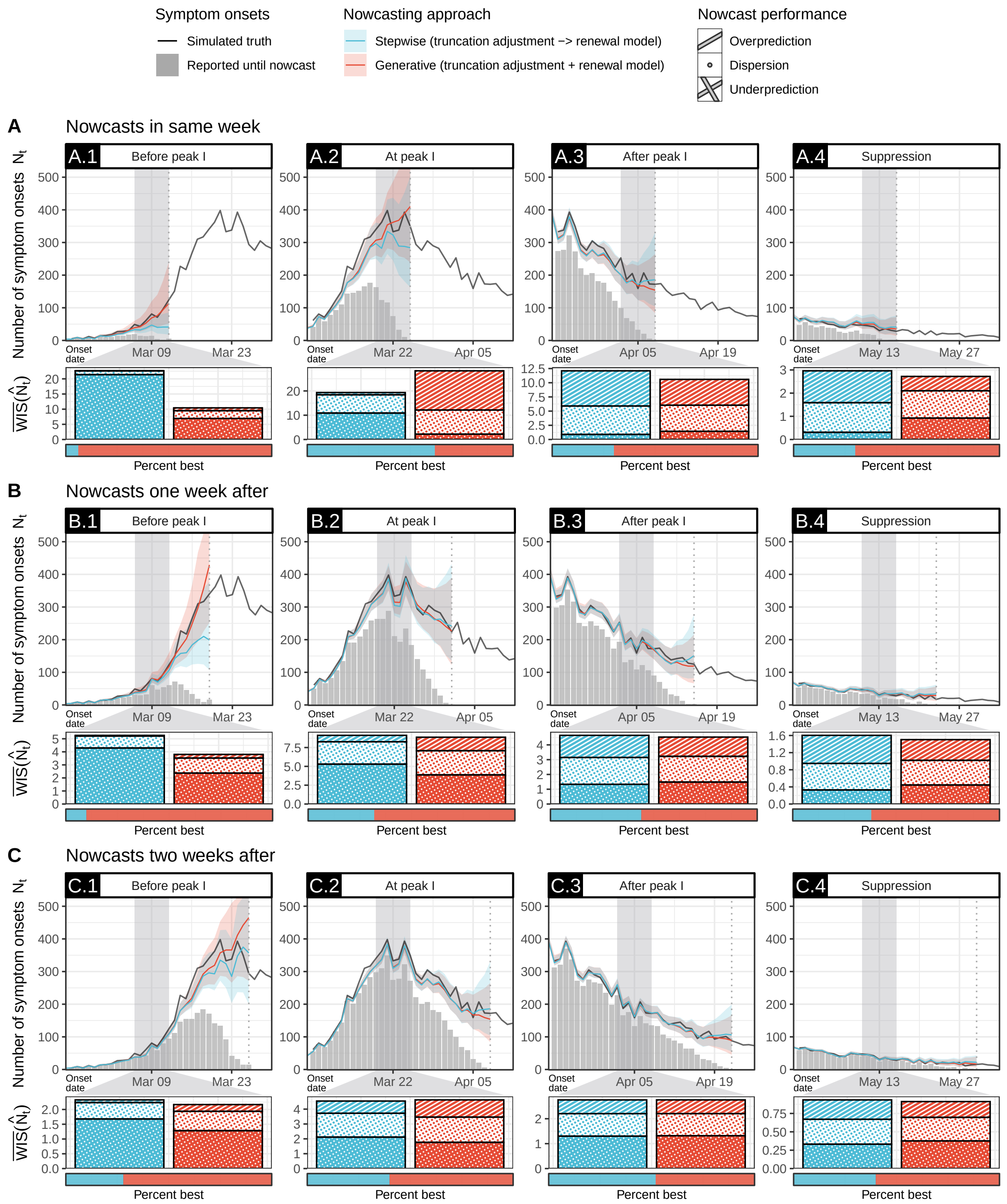

Fig N. Nowcasts of N_t on line list data of a simulated first wave scenario using different approaches of adjusting for right truncation under misspecification of the incubation period distribution (50% shorter mean). Shown are the true number of cases by symptom onset date N_t (black), the number of cases reported until the nowcast date (grey bars), and point nowcasts with 95% credible intervals (CrI) in four different phases of the epidemic wave, obtained through i) a stepwise approach using cases by date of symptom onset with a truncation adjustment step (blue), and ii) a generative approach using cases by date of symptom onset with an integrated truncation and renewal model (red). **All nowcasts assumed a misspecified incubation period distribution that had a 50% shorter mean than the true distribution.** Shown below each phase is the weighted interval score (WIS, lower is better) for N_t nowcasts of each approach during a selected week (grey shade) over 50 scenario runs (see Table L in S1 Appendix for exact figures). Colored bars show average scores, decomposed into penalties for underprediction (crosshatch), dispersion (circles), and overprediction (stripes). dispersion (circles), and overprediction (stripes). The horizontal bar below shows the percentage of times each approach achieved the lowest WIS out of 50 scenario runs, respectively. Results are shown for nowcasts made at different lags from the selected week (vertical dotted lines), i. e. at the end of the selected week (top row), one week later (middle row), and two weeks later (bottom row).

Phase	Lag [days]	Stepwise		Generative	
		$\overline{\text{WIS}}(\hat{N}_t)$	$\%^{\text{best}}$	$\overline{\text{WIS}}(\hat{N}_t)$	$\%^{\text{best}}$
Before peak I	0-6	22.65	6%	10.43	94%
	7-13	5.26	10%	3.80	90%
	14-20	2.33	28%	2.17	72%
At peak I	0-6	19.32	62%	28.28	38%
	7-13	9.12	32%	8.87	68%
	14-20	4.54	40%	4.62	60%
After peak I	0-6	12.10	30%	10.59	70%
	7-13	4.64	44%	4.53	56%
	14-20	2.75	52%	2.76	48%
Suppression	0-6	2.96	30%	2.72	70%
	7-13	1.60	38%	1.50	62%
	14-20	0.94	40%	0.91	60%

Table L. Performance of nowcasts of N_t on line list data of a simulated first wave scenario using different approaches of adjusting for right truncation under misspecification of the incubation period distribution (50% shorter mean). Shown is the performance of N_t nowcasts in four different phases of the epidemic wave, at different lags of a selected week (same week [0–6 days], one week after [7–13 days], and two weeks after [14–20 days]). Nowcasts were obtained through i) a stepwise approach using cases by date of symptom onset with a truncation adjustment step, and ii) a generative approach using cases by date of symptom onset with an integrated truncation and renewal model. **All nowcasts assumed a misspecified incubation period distribution that had a 50% shorter mean than the true distribution.** Performance is measured by the weighted interval score (WIS, lower is better), shown is the average score over 50 scenario runs ($\overline{\text{WIS}}$) and the percentage of runs in which each approach achieved the best score ($\%^{\text{best}}$).

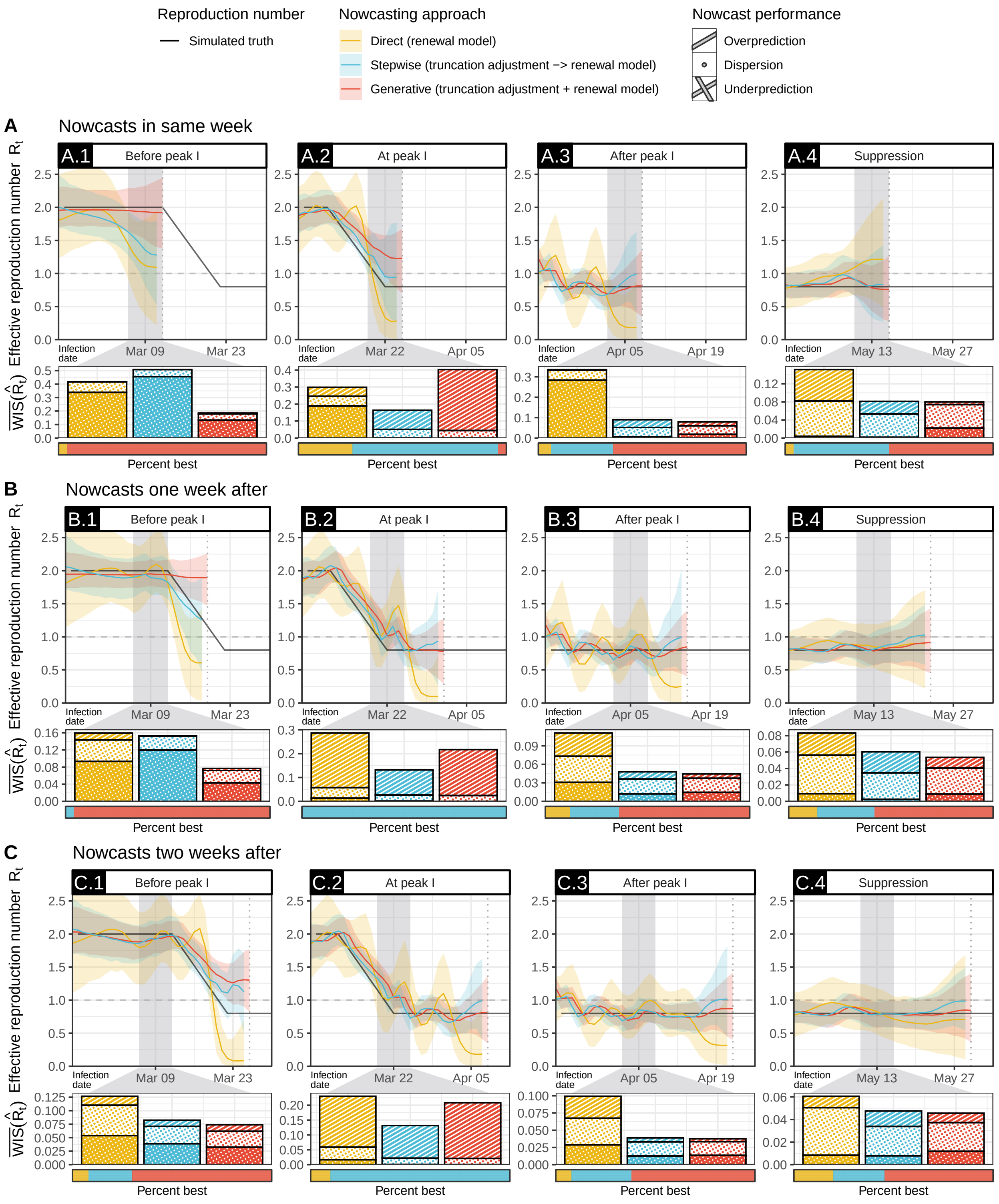

Fig O. Nowcasts of R_t on line list data of a simulated first scenario using different approaches of adjusting for right truncation under misspecification of the incubation period distribution (50% shorter mean). Shown are the true R_t (black) and point nowcasts with 95% credible intervals (CrI) in four different phases of the epidemic wave, obtained through i) a direct approach using cases by date of report with no truncation adjustment (yellow), ii) a stepwise approach using cases by date of symptom onset with a truncation adjustment step (blue), and iii) a generative approach using cases by date of symptom onset with an integrated truncation and renewal model (red). **All nowcasts assumed a misspecified incubation period distribution that had a 50% shorter mean than the true distribution.** Shown below each phase is the weighted interval score (WIS, lower is better) for R_t nowcasts of each approach during a selected week (grey shade) over 50 scenario runs (see Table M in S1 Appendix for exact figures). Colored bars show average scores, decomposed into penalties for underprediction (crosshatch), dispersion (circles), and overprediction (stripes). dispersion (circles), and overprediction (stripes). The horizontal bar below shows the percentage of times each approach achieved the lowest WIS out of 50 scenario runs, respectively. Results are shown for nowcasts made at different lags from the selected week (vertical dotted lines), i. e. at the end of the selected week (top row), one week later (middle row), and two weeks later (bottom row).

Phase	Lag [days]	Direct		Stepwise		Generative	
		$\overline{\text{WIS}}(R_t)$	$\%^{\text{best}}$	$\overline{\text{WIS}}(R_t)$	$\%^{\text{best}}$	$\overline{\text{WIS}}(R_t)$	$\%^{\text{best}}$
Before peak I	0-6	0.42	4%	0.51	0%	0.18	96%
	7-13	0.16	0%	0.15	4%	0.08	96%
	14-20	0.13	8%	0.08	22%	0.07	70%
At peak I	0-6	0.30	26%	0.16	70%	0.40	4%
	7-13	0.29	0%	0.13	100%	0.22	0%
	14-20	0.23	10%	0.13	90%	0.21	0%
After peak I	0-6	0.34	6%	0.09	30%	0.08	64%
	7-13	0.11	12%	0.05	24%	0.04	64%
	14-20	0.10	8%	0.04	30%	0.04	62%
Suppression	0-6	0.15	4%	0.08	46%	0.08	50%
	7-13	0.08	14%	0.06	28%	0.05	58%
	14-20	0.06	20%	0.05	26%	0.05	55%

Table M. Performance of nowcasts of R_t on line list data of a simulated first wave scenario using different approaches of adjusting for right truncation under misspecification of the incubation period distribution (50% shorter mean). Shown is the performance of R_t nowcasts in four different phases of the epidemic wave, at different lags of a selected week (same week [0–6 days], one week after [7–13 days], and two weeks after [14–20 days]). Nowcasts were obtained through i) a direct approach using cases by date of report with no truncation adjustment, ii) a stepwise approach using cases by date of symptom onset with a truncation adjustment step, and iii) a generative approach using cases by date of symptom onset with an integrated truncation and renewal model. **All nowcasts assumed a misspecified incubation period distribution that had a 50% shorter mean than the true distribution.** Performance is measured by the weighted interval score (WIS, lower is better), shown is the average score over 50 scenario runs ($\overline{\text{WIS}}$) and the percentage of runs in which each approach achieved the best score ($\%^{\text{best}}$).

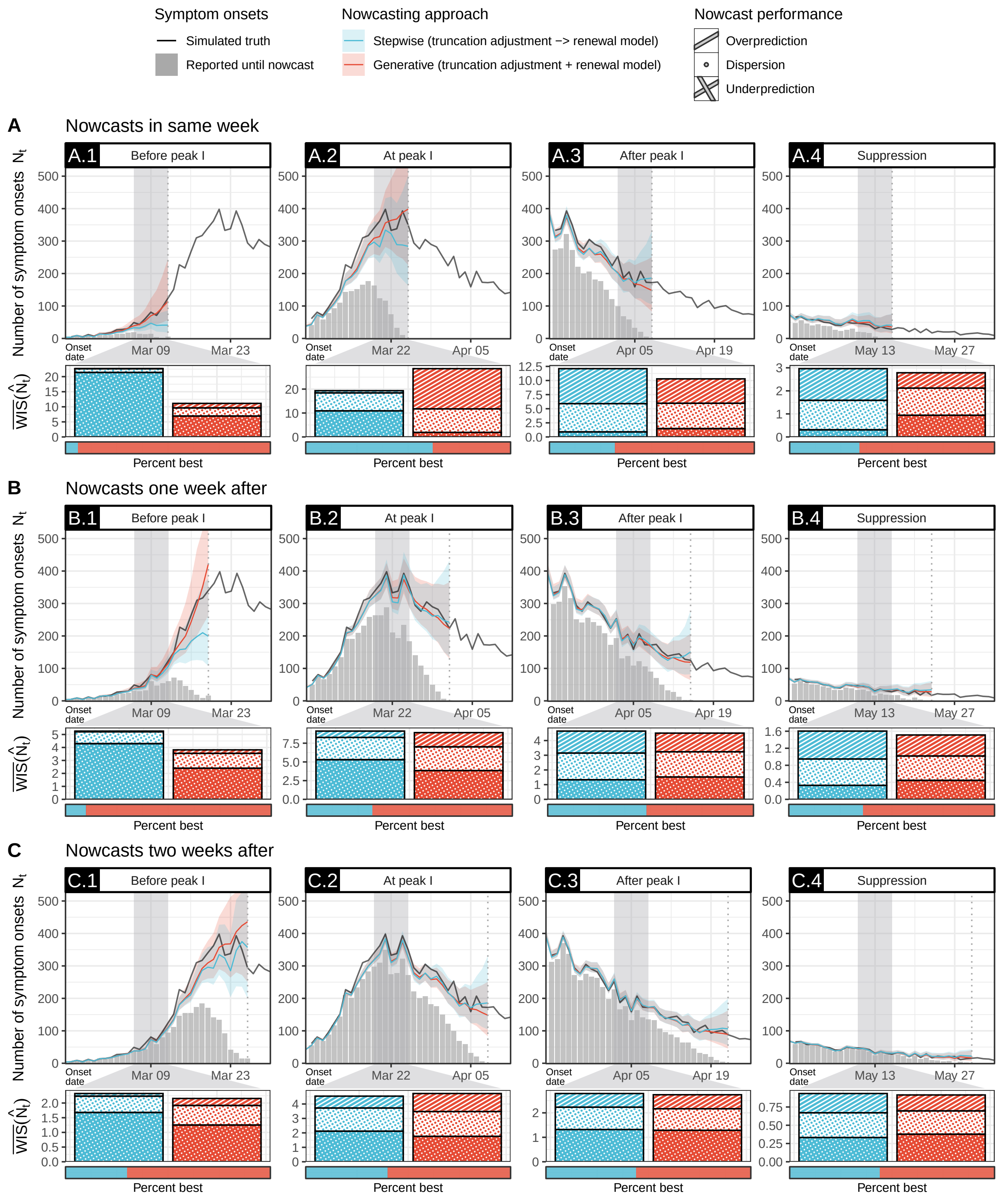

Fig P. Nowcasts of N_t on line list data of a simulated first wave scenario using different approaches of adjusting for right truncation under misspecification of the incubation period distribution (50% longer mean). Shown are the true number of cases by symptom onset date N_t (black), the number of cases reported until the nowcast date (grey bars), and point nowcasts with 95% credible intervals (CrI) in four different phases of the epidemic wave, obtained through i) a stepwise approach using cases by date of symptom onset with a truncation adjustment step (blue), and ii) a generative approach using cases by date of symptom onset with an integrated truncation and renewal model (red). **All nowcasts assumed a misspecified incubation period distribution that had a 50% longer mean than the true distribution.** Shown below each phase is the weighted interval score (WIS, lower is better) for N_t nowcasts of each approach during a selected week (grey shade) over 50 scenario runs (see Table N in S1 Appendix for exact figures). Colored bars show average scores, decomposed into penalties for underprediction (crosshatch), dispersion (circles), and overprediction (stripes). dispersion (circles), and overprediction (stripes). The horizontal bar below shows the percentage of times each approach achieved the lowest WIS out of 50 scenario runs, respectively. Results are shown for nowcasts made at different lags from the selected week (vertical dotted lines), i. e. at the end of the selected week (top row), one week later (middle row), and two weeks later (bottom row).

Phase	Lag [days]	Stepwise		Generative	
		$\overline{\text{WIS}}(\hat{N}_t)$	$\%^{\text{best}}$	$\overline{\text{WIS}}(\hat{N}_t)$	$\%^{\text{best}}$
Before peak I	0-6	22.65	6%	11.13	94%
	7-13	5.26	10%	3.81	90%
	14-20	2.33	30%	2.15	70%
At peak I	0-6	19.32	62%	28.53	38%
	7-13	9.12	32%	8.91	68%
	14-20	4.54	40%	4.73	60%
After peak I	0-6	12.10	32%	10.29	68%
	7-13	4.64	48%	4.50	52%
	14-20	2.79	44%	2.74	56%
Suppression	0-6	2.96	34%	2.79	66%
	7-13	1.60	36%	1.51	64%
	14-20	0.94	44%	0.92	56%

Table N. Performance of nowcasts of N_t on line list data of a simulated first wave scenario using different approaches of adjusting for right truncation under misspecification of the incubation period distribution (50% longer mean). Shown is the performance of N_t nowcasts in four different phases of the epidemic wave, at different lags of a selected week (same week [0–6 days], one week after [7–13 days], and two weeks after [14–20 days]). Nowcasts were obtained through i) a stepwise approach using cases by date of symptom onset with a truncation adjustment step, and ii) a generative approach using cases by date of symptom onset with an integrated truncation and renewal model. **All nowcasts assumed a misspecified incubation period distribution that had a 50% longer mean than the true distribution.** Performance is measured by the weighted interval score (WIS, lower is better), shown is the average score over 50 scenario runs ($\overline{\text{WIS}}$) and the percentage of runs in which each approach achieved the best score ($\%^{\text{best}}$).

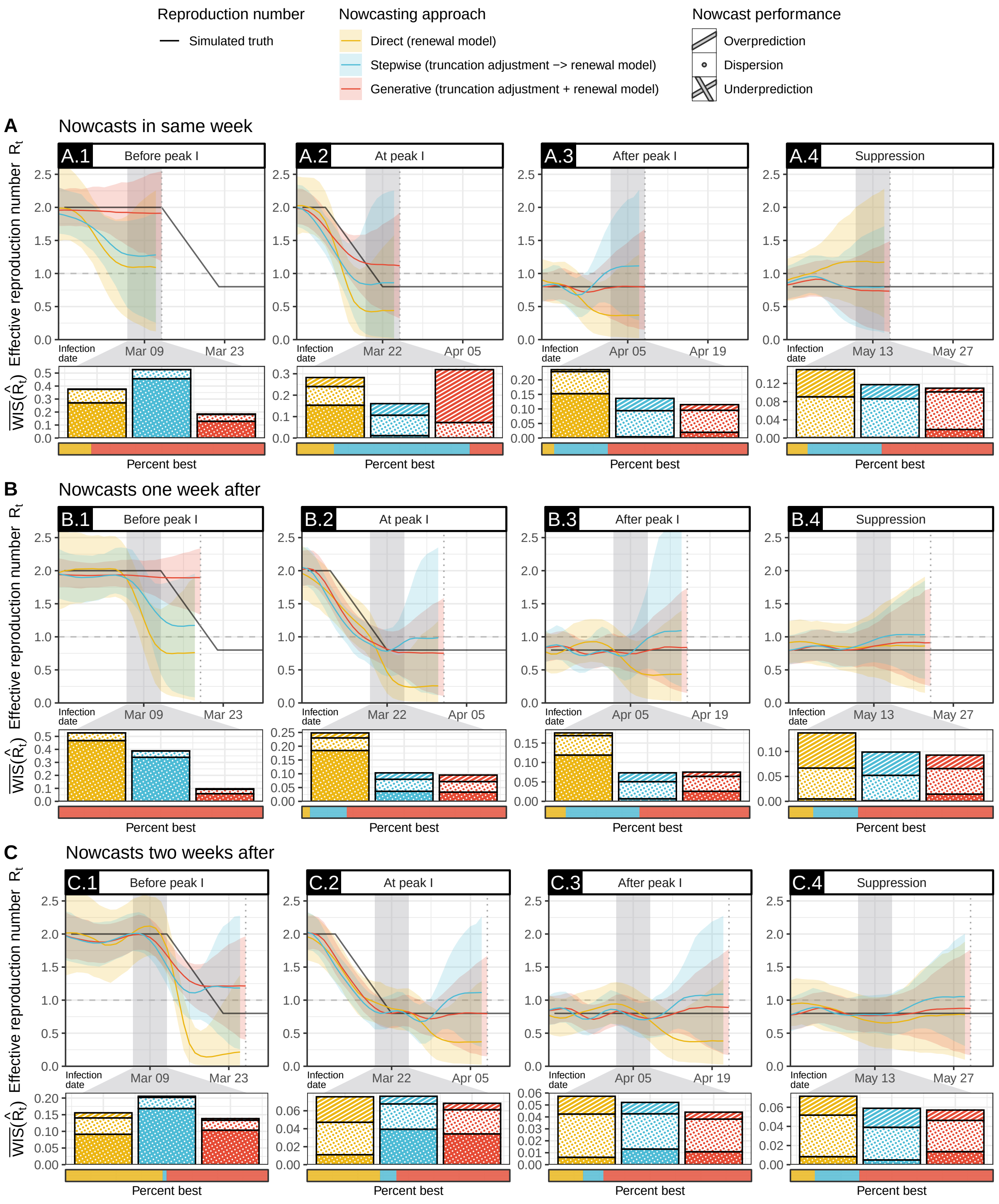

Fig Q. Nowcasts of R_t on line list data of a simulated first scenario using different approaches of adjusting for right truncation under misspecification of the incubation period distribution (50% longer mean). Shown are the true R_t (black) and point nowcasts with 95% credible intervals (CrI) in four different phases of the epidemic wave, obtained through i) a direct approach using cases by date of report with no truncation adjustment (yellow), ii) a stepwise approach using cases by date of symptom onset with a truncation adjustment step (blue), and iii) a generative approach using cases by date of symptom onset with an integrated truncation and renewal model (red). **All nowcasts assumed a misspecified incubation period distribution that had a 50% longer mean than the true distribution.** Shown below each phase is the weighted interval score (WIS, lower is better) for R_t nowcasts of each approach during a selected week (grey shade) over 50 scenario runs (see Table O in S1 Appendix for exact figures). Colored bars show average scores, decomposed into penalties for underprediction (crosshatch), dispersion (circles), and overprediction (stripes). dispersion (circles), and overprediction (stripes). The horizontal bar below shows the percentage of times each approach achieved the lowest WIS out of 50 scenario runs, respectively. Results are shown for nowcasts made at different lags from the selected week (vertical dotted lines), i.e. at the end of the selected week (top row), one week later (middle row), and two weeks later (bottom row).

Phase	Lag [days]	Direct		Stepwise		Generative	
		$\overline{\text{WIS}}(R_t)$	$\%^{\text{best}}$	$\overline{\text{WIS}}(R_t)$	$\%^{\text{best}}$	$\overline{\text{WIS}}(R_t)$	$\%^{\text{best}}$
Before peak I	0-6	0.38	16%	0.53	0%	0.18	84%
	7-13	0.53	0%	0.39	0%	0.10	100%
	14-20	0.16	48%	0.20	2%	0.14	50%
At peak I	0-6	0.28	18%	0.16	66%	0.32	16%
	7-13	0.25	4%	0.10	18%	0.10	78%
	14-20	0.08	36%	0.08	8%	0.07	56%
After peak I	0-6	0.23	6%	0.14	26%	0.12	68%
	7-13	0.18	10%	0.07	36%	0.07	54%
	14-20	0.06	17%	0.05	10%	0.04	73%
Suppression	0-6	0.15	10%	0.12	36%	0.11	54%
	7-13	0.14	12%	0.10	22%	0.09	66%
	14-20	0.07	12%	0.06	22%	0.06	66%

Table O. Performance of nowcasts of R_t on line list data of a simulated first wave scenario using different approaches of adjusting for right truncation under misspecification of the incubation period distribution (50% longer mean). Shown is the performance of R_t nowcasts in four different phases of the epidemic wave, at different lags of a selected week (same week [0–6 days], one week after [7–13 days], and two weeks after [14–20 days]). Nowcasts were obtained through i) a direct approach using cases by date of report with no truncation adjustment, ii) a stepwise approach using cases by date of symptom onset with a truncation adjustment step, and iii) a generative approach using cases by date of symptom onset with an integrated truncation and renewal model. **All nowcasts assumed a misspecified incubation period distribution that had a 50% longer mean than the true distribution.** Performance is measured by the weighted interval score (WIS, lower is better), shown is the average score over 50 scenario runs ($\overline{\text{WIS}}$) and the percentage of runs in which each approach achieved the best score ($\%^{\text{best}}$).

E Application to COVID-19 in Switzerland

E.1 Hospitalization line list data

Hospitalization line list data of patients who tested positive for COVID-19 was provided in anonymized form by the Swiss Federal Office of Public Health (FOPH). Cases were stratified by date of symptom onset, date of hospitalization, and date of report of the hospitalization to FOPH. When nowcasting, it is essential to use the date when a case effectively became available for analysis, which was the date of report of the hospitalization in our case. If we instead used the date of hospitalization as report date, any right truncation resulting from the delay between hospitalization and reporting of the hospitalization would be neglected, and the nowcasts would still be downward biased. The modeled delays in this study therefore implicitly included both the delay between symptom onset and hospitalization and the delay between hospitalization and reporting of the hospitalization. The date of hospitalization itself was not explicitly modeled. Fig R in S1 Appendix shows the number of cases over time by date of report of the hospitalization, stratified by cases with known and with missing symptom onset date. The weekly moving average percentage of cases with missing symptom onset date in the line list varied between 16% and 63%. Fig S in S1 Appendix shows empirical delays between symptom onset and report of hospitalization in different time periods of the COVID-19 epidemic in Switzerland. The assumed maximum delay of 56 days covered 97.03% of cases. Of these, the mean (standard deviation) of delays was 10.12 (9.23) days from March 1, 2020 – May 31, 2020, 11.05 (10.91) days from June 1, 2020 – Sep 31, 2020, and 8.19 (6.85) days from Oct 1, 2020 – March 31, 2021, respectively.

E.2 Time-varying epidemiological parameters

To account for the introduction and spread of the alpha variant of SARS-CoV-2 in Switzerland, we used different incubation period and generation interval distributions for the nowcasting models over time (parameters as provided in main text). All distributions were discretized and truncated at 21 days. To model the transition from introduction to dominance (i. e. over 90% of total sequences per week) of the variant B.1.1.7 (20I) alpha, we used the time period from December 21, 2020 – March 29, 2021 [24]. During the transition phase, we used a mixture of the distribution for the wildtype variant and the distribution for the alpha variant, increasing the weight for the alpha variant from 0

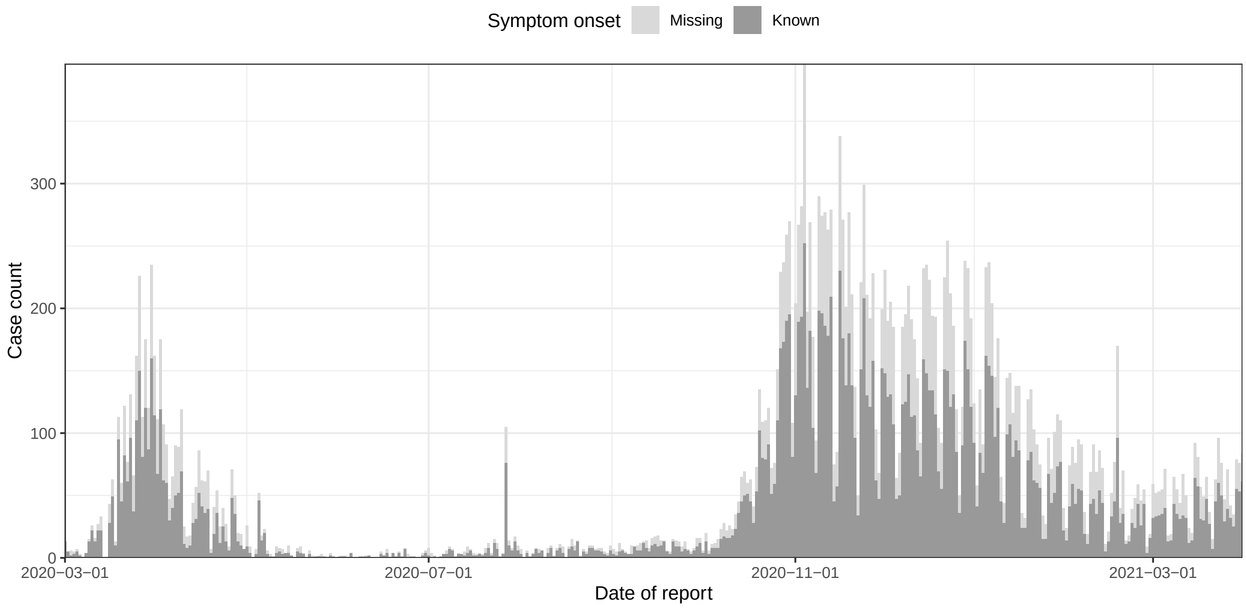

Fig R. Cases of hospitalization with COVID-19 in Switzerland, March 1, 2020 – March 31, 2021. Shown is the number of hospitalized patients who tested positive for COVID-19 by date of report of the hospitalization, during the first and second wave of COVID-19 in Switzerland. The case counts are disaggregated into cases with known (dark grey bars) and missing (light grey bars) symptom onset date.

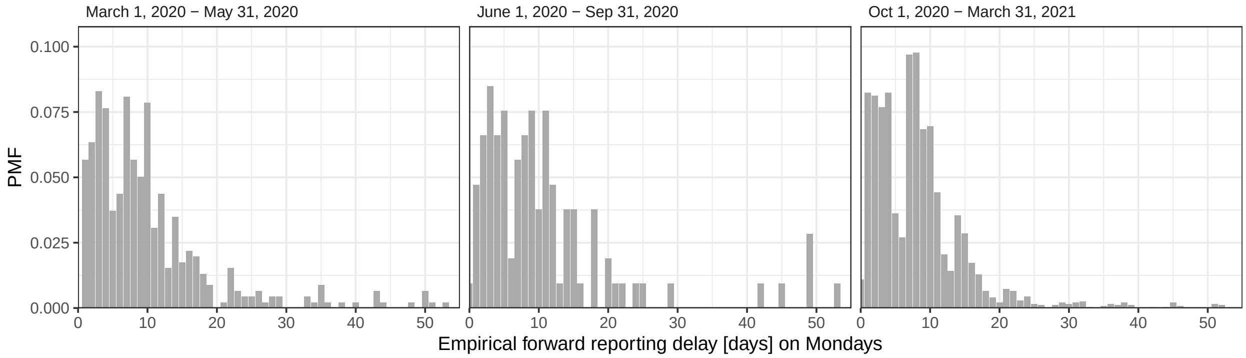

Fig S. Empirical reporting delay distribution of cases hospitalized with COVID-19 in Switzerland, March 1, 2020 – March 31, 2021. Shown is the empirical distribution of the forward delay from symptom onset to report of hospitalization during different time periods of the first and second wave of COVID-19 in Switzerland. To highlight the patterns of weekly seasonality, only cases with symptom onset on a Monday were included.

to 1 according to a logistic function. Note that for simplicity, we did not include the time-varying incubation period and generation interval distributions explicitly in the renewal model but simply supplied different parameters to the nowcasting models over time.

E.3 Overdispersion

We used a Negative Binomial likelihood for the COVID-19 data from Switzerland to account for overdispersion in case counts. Fig T in S1 Appendix shows our weakly informative prior for the transformed overdispersion parameter $\frac{1}{\sqrt{\phi}}$ (as described in Supplement B.6) and its posterior distribution from an exemplary nowcast. As can be seen, the degree of overdispersion is strongly informed by the data.

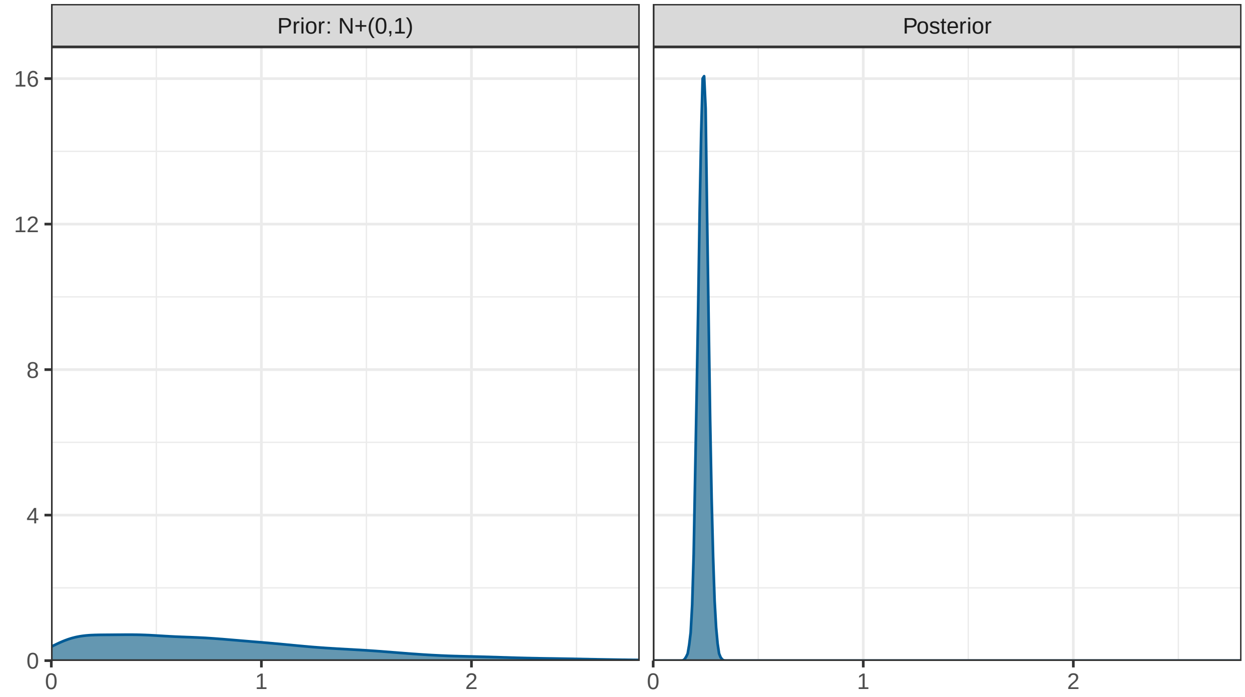

Fig T. Comparison of prior and posterior for overdispersion in COVID-19 hospitalization data. Shown is the prior and posterior density of the transformed overdispersion parameter $\frac{1}{\sqrt{\phi}}$ for the number of hospitalizations with COVID-19 in Switzerland, estimated from a nowcast on March 28, 2022 using the fully generative approach.

References

1. McGough SF, Johansson MA, Lipsitch M, Menzies NA. Nowcasting by Bayesian Smoothing: A flexible, generalizable model for real-time epidemic tracking. PLOS Computational Biology. 2020;16(4):e1007735. doi:10.1371/journal.pcbi.1007735.

2. Bastos LS, Economou T, Gomes MFC, Villela DAM, Coelho FC, Cruz OG, et al. A modelling approach for correcting reporting delays in disease surveillance data. Statistics in Medicine. 2019;38(22):4363–4377. doi:10.1002/sim.8303.

3. Günther F, Bender A, Katz K, Küchenhoff H, Höhle M. Nowcasting the COVID-19 pandemic in Bavaria. Biometrical Journal. 2021;63(3):490–502. doi:10.1002/bimj.202000112.

4. Hyndman RJ, Athanasopoulos G. Forecasting: Principles and Practice. 2nd ed. OTexts; 2018.

5. Dugas C, Bengio Y, Bélisle F, Nadeau C, Garcia R. Incorporating Second-Order Functional Knowledge for Better Option Pricing. In: Advances in Neural Information Processing Systems. vol. 13. MIT Press; 2000.

6. Sharma M, Mindermann S, Brauner JM, Leech G, Stephenson AB, Kulveit J, et al. How Robust are the Estimated Effects of Nonpharmaceutical Interventions against COVID-19? Advances in Neural Information Processing Systems. 2020;33:12175–12186.

7. Scott JA, Gandy A, Mishra S, Unwin J, Flaxman S, Bhatt S. Epidemia: Modeling of Epidemics Using Hierarchical Bayesian Models; 2020. Available from: https://imperialcollegelondon.github.io/epidemia/index.html.

8. Gostic KM, McGough L, Baskerville EB, Abbott S, Joshi K, Tedijanto C, et al. Practical considerations for measuring the effective reproductive number, Rt. PLOS Computational Biology. 2020;16(12):e1008409. doi:10.1371/journal.pcbi.1008409.

9. Fraser C. Estimating Individual and Household Reproduction Numbers in an Emerging Epidemic. PLOS ONE. 2007;2(8):e758. doi:10.1371/journal.pone.0000758.

10. Cori A, Ferguson NM, Fraser C, Cauchemez S. A new framework and software to estimate time-varying reproduction numbers during epidemics. American Journal of Epidemiology. 2013;178(9):1505–1512. doi:10.1093/aje/kwt133.

11. Bhatt S, Ferguson N, Flaxman S, Gandy A, Mishra S, Scott JA. Semi-Mechanistic Bayesian Modeling of COVID-19 with Renewal Processes. ArXiv [Preprint]. 2020;2012.00394.

12. Banholzer N, van Weenen E, Lison A, Cenedese A, Seeliger A, Kratzwald B, et al. Estimating the Effects of Non-Pharmaceutical Interventions on the Number of New Infections with COVID-19 during the First Epidemic Wave. PLOS ONE. 2021;16(6):e0252827.

13. Höhle M, an der Heiden M. Bayesian nowcasting during the STEC O104:H4 outbreak in Germany, 2011. Biometrics. 2014;70:993–1002. doi:10.1111/biom.12194.

14. Cox DR. Regression Models and Life-Tables. Journal of the Royal Statistical Society: Series B (Methodological). 1972;34(2):187–202.

15. Stan development team. Prior Choice Recommendations; 2020. Available from: https://github.com/stan-dev/stan/wiki/Prior-Choice-Recommendations.

16. Stan development team. Stan Modeling Language Users Guide and Reference Manual, Version 2.31; 2022. Available from: https://mc-stan.org.

17. Gabry J, Češnovar R. type [; 2022]Available from: https://mc-stan.org/cmdstanr.

18. Geyer C. Introduction to Markov Chain Monte Carlo. Brooks S, Gelman A, Jones G, Meng XL, editors. Chapman and Hall/CRC; 2011.

19. Betancourt M. A Conceptual Introduction to Hamiltonian Monte Carlo. arXiv [preprint]. 2018;doi:10.48550/arXiv.1701.02434.

20. Betancourt M. Diagnosing Suboptimal Cotangent Disintegrations in Hamiltonian Monte Carlo. arXiv [preprint]. 2016;doi:10.48550/arXiv.1604.00695.

21. Gelman A, Rubin DB, et al. Inference from iterative simulation using multiple sequences. Statistical Science. 1992;7(4):457–472.

22. Hart WS, Abbott S, Endo A, Hellewell J, Miller E, Andrews N, et al. Inference of the SARS-CoV-2 Generation Time Using UK Household Data. eLife. 2022;11:e70767.

23. Linton NM, Kobayashi T, Yang Y, Hayashi K, Akhmetzhanov AR, Jung Sm, et al. Incubation Period and Other Epidemiological Characteristics of 2019 Novel Coronavirus Infections with Right Truncation: A Statistical Analysis of Publicly Available Case Data. Journal of Clinical Medicine. 2020;9(2):538. doi:10.3390/jcm9020538.

24. Hodcroft E, Aksamentov I, Neher R, Bedford T, Hadfield J, Zuber M, et al.. CoVariants: SARS-CoV-2 Mutations and Variants of Interest; 2023. Available from: https://covariants.org/per-country?region=Switzerland.
