# Supplementary material for: Generative Bayesian modeling to nowcast the effective reproduction number from line list data with missing symptom onset dates
Source: PLoS Comput Biol. 2024 Apr 16;20(4):e1012021. doi: 10.1371/journal.pcbi.1012021 (PMC11051644; doi:10.1371/journal.pcbi.1012021)
Supplement: Attachment — Submitted filename: Review_comments_Nowcasting_PLOS_Sept_2023.pdf [file pcbi.1012021.s002.pdf]

Review comments

Generative Bayesian modeling to nowcast the effective reproduction number from line list data with missing symptom onset dates

September 2023

Overall comments

In this well-written manuscript (PCOMPBIOL-D-23-01366) Lison et al. propose a Bayesian generative model to nowcast the effective reproduction number R_t. The novelty of their model is that they integrate imputation, truncation adjustment, and R_t estimation into a single hierarchical model. They compare the performance of previous step-wise methods with their proposed joint model using simulated line list data as well as COVID-19 hospitalization data from Switzerland. I enjoyed reading the manuscript and only have a few comments expressed below.

Major comments

- I suggest writing something about *how* you compare the models in the introduction and what your major findings are and how you came to that conclusion.
  - P 4, l 58. "Based on our findings..." which are? State major findings in introduction to motivate your proposed model. When is it better and why.
- I find in a few places that results are stated but not interpreted or backed up with how it is estimated. Explain how you come to the conclusion and/or possibly refer to a figure/table. For example:
  - P 26, l 507. "we found the same qualitative differences between the generative and the stepwise approach"
  - P 27, l 524. "mostly agreed after a lag of two weeks but showed systematic differences at shorter lags"
- When comparing different models like (p 23 l 473, p 25 l 513) I would be more specific with how much and give explanations to why this is the case. Also here you could reference a figure and give some interpretations of the results.

- In Fig 2-7 there are many small plots with multiple overlaying shaded areas, lines and bars making the figures hard to read. Could you do a selection of period or Nowcast week to illustrate your main message? I suggest naming each sub-figure (A1, A2, ..., C4) and refer to the subfigures when discussing or interpreting the results. I believe that if a figure is not thoroughly explained or discussed in text that it is necessary to have it in the main article but instead could be moved to the appendix.
- A table of summarized results would be good to get an overview of the results. Reading the avg WIS from each of the sub-plots is not optimal.
- Clarify why you first write Eq 4 and then say that you use Eq 6 for all models in a new separate section. Why not directly write Eq 6?
- P 7, l 149. What is a *sufficiently* large maximum delay D? Explain how and why you choose 8 weeks.
- Are the models equally fast? What is the runtime? I would assume the joint model takes longer to run but nothing is written about the computational cost.

Minor comments

- Sentence no 2 in abstract is unclear. ..observations *of reported cases*? And what is the *original date of infection*, I believe it is simply the *date of infection*.
- In abstract l 4 from below. What is "long" reporting delays in this context?
- P 3, l 51. "...compare performance and qualitative behavior...". Please be more specific to what you mean with *compare*, and what the *performance* and *qualitative behavior* actually is.
- P 4, l 38. Reference Li & White.
- P 5, l 62. Describe and motivate models first, then refer to the scheme (Fig 1).
- P 5, l 65. Number of *reported* cases.
- P 8, l 129. N_t is not previously defined, how does it relate to $N_{d,t}$.
- P 14, l 328 & 329. Reference to WIS and CRPS.
- The "percent best" in Fig 2-5. It not specified what is meant by "best" and it is hard to see the results when its in a bar (a possibility would be to put the actual numbers there too or use a table). Also, why is is excluded in Fig 6?
- P 20, l 455. How much higher (or relative improvement) WIS .
