# Supplementary material for: Generative Bayesian modeling to nowcast the effective reproduction number from line list data with missing symptom onset dates
Source: PLoS Comput Biol. 2024 Apr 16;20(4):e1012021. doi: 10.1371/journal.pcbi.1012021 (PMC11051644; doi:10.1371/journal.pcbi.1012021)
Supplement: Attachment — Submitted filename: Reponse letter.pdf [file pcbi.1012021.s003.pdf]

Point-by-point response to reviewers

Manuscript title: "Generative Bayesian modeling to nowcast the effective reproduction number from line list data with missing symptom onset dates"

Dear Prof. Britton, dear reviewers,

Thank you for your detailed and constructive reviews on our submitted manuscript ″Generative Bayesian modeling to nowcast the effective reproduction number from line list data with missing symptom onset dates″. We welcome the opportunity to submit a thoroughly revised version of our manuscript together with this point-by-point response letter.
We greatly appreciate the positive and helpful feedback from the reviewers. In the following, we would like to summarize the most important changes that we incorporated during the revision (highlighted **in blue** in the revised manuscript):

1. **Coherence of Results section:** Based on feedback from both reviewers, we have revised our manuscript to make it easier for readers to follow the presentation of our results. This includes improved labeling of subfigures and referencing in the text, results tables with detailed performance scores, and an overview table containing a summary of our main findings.

2. **Additional sensitivity analysis:** In response to a question by reviewer #2, we have conducted a sensitivity analysis to assess the influence of using a misspecified incubation period distribution on nowcasting results.

3. **Expanded discussion of method limitations and applicability:** Based on the helpful comments by reviewer #2, we have revised and expanded our Discussion section to better address the strengths and limitations of our method from a public health perspective. In particular, we point out limitations in detecting changes in R_t and more carefully explain the advantages of generative modeling in the context of surveillance for situational awareness and short-term planning.

We believe that our improvements made in response to your feedback have significantly benefited the manuscript. Below we provide a point-by-point response to all of your comments, along with the corresponding changes made to the main text and SI Appendix. We hope to have addressed everything to your satisfaction.

Response to Reviewer #1

Overall comments

Comment 1.0: In this well-written manuscript (PCOMPBIOL-D-23-01366) Lison et al. propose a Bayesian generative model to nowcast the effective reproduction number R_t. The novelty of their model is that they integrate imputation, truncation adjustment, and R_t estimation into a single hierarchical model. They compare the performance of previous step-wise methods with their proposed joint model using simulated line list data as well as COVID-19 hospitalization data from Switzerland. I enjoyed reading the manuscript and only have a few comments expressed below.

Response 1.0: Thank you for your positive feedback and insightful comments. We are pleased that you find our manuscript valuable and have addressed your comments below.

Major comments

Comment 1.1: I suggest writing something about how you compare the models in the introduction and what your major findings are and how you came to that conclusion.

- P 4, l 58. ”Based on our findings...” which are? State major findings in introduction to motivate your proposed model. When is it better and why.

Response 1.1: Thank you for this suggestion, we have added a short description of our main findings to the introduction.

Comment 1.2: I find in a few places that results are stated but not interpreted or backed up with how it is estimated. Explain how you come to the conclusion and/or possibly refer to a figure/table. For example:

- P 26, l 507. ”we found the same qualitative differences between the generative and the stepwise approach”
- P 27, l 524. ”mostly agreed after a lag of two weeks but showed systematic differences at shorter lags”

Response 1.2: This is valuable feedback, we have revised our Results section to be more precise and have added references to corresponding figures (and subfigures) as well as result tables with the precise numbers.

Comment 1.3: When comparing different models like (p 23 l 473, p 25 l 513) I would be more specific with how much and give explanations to why this is the case. Also here you could reference a figure and give some interpretations of the results.

Response 1.3: We agree and have added more detailed explanations for the model comparisons with concrete quantities, as well as references to corresponding (sub-)figures and result tables.

Comment 1.4: In Fig 2-7 there are many small plots with multiple overlaying shaded areas, lines and bars making the figures hard to read. Could you do a selection of period or Nowcast week to illustrate your main message? I suggest naming each sub-figure (A1, A2, ..., C4) and refer to the subfigures when discussing or interpreting the results. I believe that if a figure is not thoroughly explained or discussed in text that it is necessary to have it in the main article but instead could be moved to the appendix.

Response 1.4: Thank you for your suggestion. As we prefer to have a consistent selection of phases and nowcast weeks across all result figures, we would like to preserve the current layout. However, we very much liked your idea of naming the individual sub-figures and have therefore added corresponding labels (A1, A2, …) to all figures. Using these labels, we have furthermore revised the Results section to refer to specific phases and weeks of interest when interpreting the nowcasting results. We hope this will help readers understand and interpret the different subfigures.

Comment 1.5: A table of summarized results would be good to get an overview of the results. Reading the avg WIS from each of the sub-plots is not optimal.

Response 1.5: We appreciate your suggestion and have added tables summarizing the quantitative performance of the different approaches (average WIS and percent best, i.e. percentage of times each approach achieved the lowest WIS out of 50 scenario runs) for the different subsections. We have also created an overview table that states the main qualitative findings of our evaluation.

Comment 1.6: Clarify why you first write Eq 4 and then say that you use Eq 6 for all models in a new separate section. Why not directly write Eq 6?

Response 1.6: Thank you for your question. We do not use Eq. 4 and Eq. 6 interchangeably in our models. Eq. 4 describes the likelihood of the observed numbers of cases with symptom onset on date and reporting delay d. In contrast, Eq. 6 describes our smoothing prior for the expected number of symptom onsets, which is a latent variable in our model.

Did you perhaps mean to refer to section 2.4, where we replace the Poisson likelihood from Eq. 4 with a Negative Binomial likelihood as described in Eq. 12? We only use the Negative Binomial likelihood for our analysis of real-world data from Switzerland, to account for overdispersion in the COVID-19 data. In our simulation study with synthetic data, we use the Poisson likelihood from Eq. 4 as we regard this as the basic model. Overdispersion should be added to the model if warranted by the specific data analyzed. For instance, in our analysis of COVID-19 data from Switzerland, we estimated significant overdispersion in the case counts.

In response to your comment, we revised section 2.4 to clarify that Eq. 12 is proposed as an extension of the basic model and that we use it for our analysis of real-world data. We also added a section in the appendix (SI Appendix E.3) where we show the estimated overdispersion for an exemplary nowcast and indicate the possible choice between the Poisson and Negative Binomial likelihood in the model specifications in SI Appendix B.

Comment 1.7: P 7, l 149. What is a sufficiently large maximum delay D? Explain how and why you choose 8 weeks.

Response 1.7: This is indeed an important aspect, and we have expanded our explanation of the maximum delay choice in response to your comment. Most importantly, we argue that the modeled delay distribution should almost fully cover the true delay distribution and only exclude potential outliers. In practice, the choice of the maximum delay can be informed by already observed delays (e.g. the 95% empirical quantile of delays), however, we stress that it should ultimately be based on domain knowledge about the reporting system, especially in the early phase of an outbreak. For our retrospective analysis of COVID-19 data in Switzerland, we chose a maximum delay of 8 weeks as this covered more than 95% of empirical delays in our data and was consistent with our knowledge of the hospital reporting to the Federal Office of Public Health. In contrast, for a non-retrospective analysis during a novel outbreak, we would initially base our choice of the maximum delay rather on experience with previous public health emergencies, and add a safety margin of several weeks. Of note, choosing a larger maximum delay than necessary increases the estimation runtime (for all approaches), but does not bias our estimates of the delay distribution.

Comment 1.8: Are the models equally fast? What is the runtime? I would assume the joint model takes longer to run but nothing is written about the computational cost.

Response 1.8: We agree that this is important information and have added a corresponding section with details about the runtimes of the different approaches to the Results (see section 3.1.6). In short, the joint model has a longer overall runtime than the individual models fitted in the stepwise approach, however, the relative speed of the approaches depends on the number of resampling iterations used by the stepwise approach. The resampling is required to account for the uncertainty of the truncation adjustment in the stepwise approach and involves repeated application of the R_t estimation step. To match the number of posterior N_t samples of the generative approach, the stepwise approach would need 4000 resampling iterations, however, this is likely more than is required for sufficient uncertainty quantification. For example, we used a much smaller number of 50 resampling iterations in our main analysis, and here the stepwise approach was 2-3x faster than the generative approach. However, at a higher number of 200 resampling iterations, the stepwise approach would already have a similar runtime as the generative approach. Here a difficulty is that the number of resampling iterations required by the stepwise approach to accurately represent the uncertainty of the truncation adjustment step may vary with the scenario and delay distribution, and is difficult to predict in advance.

Minor comments

Comment 1.9: Sentence no 2 in abstract is unclear. ..observations of reported cases? And what is the original date of infection, I believe it is simply the date of infection.

Response 1.9: We understand this was rather unclear and have revised the sentence. It now reads: “Timely estimates of R_t can be obtained from reported cases counted by their date of symptom onset, which is generally closer to the time of infection than the date of report.”

Comment 1.10: In abstract l 4 from below. What is ”long” reporting delays in this context?

Response 1.10: Thank you, we now provide more detail in the abstract, i.e. “reporting delays realistic for hospitalization data (50% of reports delayed by more than a week)”. We also explain this in the methods: “Under such a reporting delay, approximately 50% of hospitalizations are reported within one week after symptom onset, and 80% after two weeks.”

Comment 1.11: P 3, l 51. ”...compare performance and qualitative behavior...”. Please be more specific to what you mean with compare, and what the performance and qualitative behavior actually is.

Response 1.11: Good point, we now say more clearly that we study “uncertainty, over- and underprediction, and probabilistic forecast scores of nowcasts”. We also state our main findings at the end of the Introduction, as per your earlier comment 1.1.

Comment 1.12: P 4, l 38. Reference Li & White.

Response 1.12: Thank you, we have moved the citation further forward in the sentence.

Comment 1.13: P 5, l 62. Describe and motivate models first, then refer to the scheme (Fig 1).

Response 1.13: This is a good suggestion, we have moved the reference to later in the paragraph and refer to specific parts of the scheme (A, B, and C) where suitable.

Comment 1.14: P 5, l 65. Number of reported cases.

Response 1.14: Good point, we have updated this.

Comment 1.15: P 8, l 129. N_t is not previously defined, how does it relate to $N_{d,t}$.

Response 1.15: N_t is the total case count by date of symptom onset *over all delays*, i.e. $N_t = \sum_{d=0}^{D} N_{t,d}$. It can only be observed after the maximum reporting delay D. We have revised the description to be more accurate and also provided the formula for N_t in the manuscript.

Comment 1.16: P 14, l 328 & 329. Reference to WIS and CRPS.

Response 1.16: Thank you, we have added references to Bracher et al. (2021) and Gneiting and Raftery (2007).

Comment 1.17: The ”percent best” in Fig 2-5. It not specified what is meant by ”best” and it is hard to see the results when its in a bar (a possibility would be to put the actual numbers there too or use a table). Also, why is is excluded in Fig 6?

Response 1.17: The horizontal bar shows the percentage of times each approach achieved the lowest WIS out of 50 scenario runs, respectively. In response to your comment, we have revised the figure caption to explain this more clearly, and have added the exact percentages to a result

table as per your earlier suggestion in comment 1.5.

In Fig. 6, no “percent best” score over repeated scenario runs can be computed, as we evaluate nowcasting performance on real-world data in this section. The WIS scores shown in the vertical bar plots of Fig. 6 indicate which approach performed best in a given phase of the COVID-19 pandemic.

Comment 1.18: P 20, l 455. How much higher (or relative improvement) WIS .

Response 1.18: We have added the information and a reference to the corresponding result table to the manuscript.

Response to Reviewer #2

Comment 2.0: In this manuscript Lison et al. propose a method to estimate the instantaneous effective reproductive number from surveillance line-list data of COVID-19 using a bayesian framework and MCMC based on daily incidence over time. The method aims to solve two major problems in communicable disease near-real time surveillance, namely missingness and right truncation, together with estimation of R_eff. The integrative method is proposed as an alternative to stepwise approaches, aiming to improve the propagation of uncertainty. They further test their method using in silico and empirical data and compare its performance with a representative stepwise approach. The proposed method requires prior estimates of the serial interval/incubation period, unlike other recently proposed methods (Dai et al. 2023). The study is written in a clear manner and the authors explain well the formulated model and the validation steps. Also, the authors provide an R package extension for implementation for real-time surveillance.

Response 2.0: Thank you for this on-point summary of our work and your positive and constructive feedback. We found your comments highly valuable for improving the presentation and discussion of our method. We have also conducted a sensitivity analysis based on your suggestions. Please find our point-by-point response to your comments below.

Comment 2.1: There are some points that might need to be addressed, particularly concerning the limitations of the method when used in public health surveillance. In the following, this reviewer provides with a series of comments that mainly focus on the potential usefulness of the approach:

Line 95. This is a key assumption (constant ascertainment rates) which is likely not true most of the time in reality, particularly in the first stage of a pandemic/outbreak. Authors could test the performance of the methods (integrated vs. stepwise) under violations of this assumption. Otherwise, as this is a limitation that is well known but not easily addressed with statistical approaches, it would be reasonable to highlight it within the limitations in the discussion (although it is well remarked in the methods, later line 102).

Response 2.1: We agree that constant ascertainment is a critical assumption that can lead to bias in R_t estimates if violated. Importantly, this limitation is not specific to nowcasting methods. It generally applies to methods that estimate R_t from reporting data and has been documented in many previous studies (e.g. Cori et al., Gostic et al., Abbott et al., Huisman et al.).

In our Methods and Discussion sections, we have highlighted the importance of this limitation. In the discussion, we write: "...we assumed a fixed ascertainment proportion $\rho_t=\rho$ over time to ensure identifiability. Under this assumption, exact knowledge of the ascertainment proportion is not essential because a misspecification of ρ does not bias R_t and distorts uncertainty quantification only in extreme cases. It thus allows us to obtain R_t estimates from hospitalization data that are indicative of population-wide transmission, but will lead to bias in periods where ρ_t changes. This is particularly important when there are differences in reporting delays or ascertainment proportions for cases from distinct subpopulations, e.g. age groups. In such a case, it can be necessary to add group structure to the renewal and reporting delay components of the model, to avoid bias from differing transmission dynamics between the subpopulations. Moreover, time-varying ascertainment proportions may also estimated by fitting to several data sources, e.g. hospitalizations and deaths, simultaneously." As we remark in our Methods section, the bias will be small but continuous for slow, constant changes in the ascertainment proportion, and bigger but only temporary for incidental, abrupt changes.

References:

- Cori, A., Ferguson, N. M., Fraser, C. & Cauchemez, S. A new framework and software to estimate time-varying reproduction numbers during epidemics. American Journal of Epidemiology 178, 1505–1512 (2013).
- Gostic, K. M. et al. Practical considerations for measuring the effective reproductive number, Rt. PLOS Computational Biology 16, e1008409 (2020).
- Abbott, S. et al. Estimating the time-varying reproduction number of SARS-CoV-2 using national and subnational case counts. Wellcome Open Research 5, 112 (2020).
- Huisman, J. S. et al. Estimation and worldwide monitoring of the effective reproductive number of SARS-CoV-2. eLife 11, e71345 (2022).

Comment 2.2: Line 97 assuming a prior incubation period, although obviously useful for applying the method, makes the model less useful for outbreaks of emerging pathogens (as they are typically not well understood in the early stages). Would be interesting to see how deviations from this assumption (i.e. misspecified incubation period) affects nowcasting.

Response 2.2: Thank you, this is a valid and important point: From a theoretical point of view, nowcasts for N_t in the stepwise approach are independent of the assumed incubation period since the incubation period is not used during the truncation adjustment step. In the generative approach, however, nowcasts for N_t are estimated jointly with the reproduction number R_t and thus also depend on the assumed incubation period.

We therefore agree that it is valuable to assess the dependence on the assumed incubation period distribution and have conducted a corresponding sensitivity analysis. Here we misspecified the incubation period distribution to either have a 50% shorter or a 50% longer

mean than the true distribution from the main analysis. We found that while this misspecification led to worse performance of R_t nowcasts for all approaches, it did not change the performance of the N_t nowcasts, neither for the stepwise nor for the generative approach. This is an important finding as the generative approach still provided unbiased nowcasts of the exponential growth trend, indicating that it is somewhat robust to a misspecification of the incubation period distribution. We interpret this as evidence that even in the case of a misspecified incubation period distribution, the renewal model as used in the generative approach serves as a useful prior for the incidence curve, in particular during phases of exponential growth or decline. Moreover, our observation that nowcasts for R_t had a reduced performance for all approaches was expected due to the resulting temporal inaccuracy of R_t estimates (see Gostic et al.). The relative performance of the approaches remained as in our main analysis. The results of our sensitivity analysis are presented in a new subsection 3.1.5 of our manuscript.

References:

- Gostic, K. M. et al. Practical considerations for measuring the effective reproductive number, Rt. PLOS Computational Biology 16, e1008409 (2020).

Comment 2.3: Line 183. This might be an important claim for practical implementation although it is difficult to understand how this might play a role in surveillance systems. Can the authors provide more details on how this cost can impact routine surveillance?

Response 2.3: Thank you for your question, we apologize for a potential ambiguity in our statement about the runtimes of the stepwise approach. Here our intention was to point out that due to the need for resampling (and therefore repeated fitting of the R_t model), the R_t estimation step made up a considerable share of the overall runtime of the stepwise approach (55.4% for 50 resampling iterations, 83.3% for 200 resampling iterations). On the other hand, the joint model fitted in the generative approach has a longer runtime due to its higher complexity. Thus, overall, the relative speed of the different approaches depends on the number of resampling iterations used by the stepwise approach: the stepwise approach was faster than the generative approach when 50 resampling iterations were used, but would be slower if more than 200 resampling iterations are used.

In response to your comment and another comment by reviewer #1, we have added a section with details about the runtimes of the different approaches to the Results (see section 3.1.6). We have also revised the sentence in former Line 183 to avoid ambiguity.

Comment 2.4: Lines 213-244. Congratulations for this paragraph. This is a commonly misunderstood problem that is well explained here.

Response 2.4: Thank you, we highly appreciate your positive feedback.

Comment 2.5: Can authors provide more details on the pooled overdispersion parameterization and robustness of the approach? Model results might be sensitive to the prior here.

Response 2.5: The modeling of overdispersion via a pooled overdispersion parameter has been applied in earlier work for nowcasting and related applications (e.g. Günther et al.,

McGough et al., Flaxman et al., Bhatt et al.,), and was often used for case count data during the COVID-19 pandemic. We agree that a strong prior on the overdispersion parameter could influence nowcasts when there is not sufficient data to overwrite the prior. To avoid this, we used a standard half-normal prior on the transformed overdispersion parameter 1/sqrt(phi) as generally recommended by stan (see Stan development team 2020) and detailed in SI Appendix B6. This weakly informative prior mostly serves as a regularization for model fitting and avoids placing too much prior probability mass on large degrees of overdispersion.

In response to your question, we have added a section in SI Appendix E.3 where we show both the prior and the posterior density for the overdispersion parameter in our application to the COVID-19 pandemic in Switzerland. Here we found that the estimated overdispersion was strongly informed by the provided case data and differed substantially from our flat prior. Based on these results, we are confident that our modeling of overdispersion is robust under the requirement that a weakly informative prior as proposed above is used.

References:

- Günther, F., Bender, A., Katz, K., Küchenhoff, H. & Höhle, M. Nowcasting the COVID-19 pandemic in Bavaria. Biometrical Journal 63, 490–502 (2021).
- McGough, S. F., Johansson, M. A., Lipsitch, M. & Menzies, N. A. Nowcasting by Bayesian Smoothing: A flexible, generalizable model for real-time epidemic tracking. PLOS Computational Biology 16, e1007735 (2020).
- Flaxman, S. et al. Estimating the effects of non-pharmaceutical interventions on COVID-19 in Europe. Nature 584, 257–261 (2020).
- Bhatt, S. et al. Semi-Mechanistic Bayesian Modeling of COVID-19 with Renewal Processes. arXiv:2012.00394 [stat] (2020).
- Stan development team. Prior Choice Recommendations. (2020).

Comment 2.6: Line 306. This seems like a strong assumption with no reference/clear justification

Response 2.6: We apologize for not having provided a clear explanation for our choice of the hospitalization probability in our simulations. Our choice of 2% hospitalization probability is based on a seroprevalence study from late 2020 in Geneva, Switzerland (Stringhini et al.), which estimated that each confirmed SARS-CoV-2 case represented 2.3-3.1 (95% CrI) actual infections in the community. Moreover, according to the line list data provided to us by the Federal Office of Public Health, the case-hospitalization proportion was 2.5-5.7% (95% empirical quantile) during the second COVID-19 wave in Switzerland. Therefore, we estimated the infection-hospitalization proportion to be between 0.8% and 2.5%, and used 2% for simplicity. This is also in line with a study from Indiana, US, which estimated an IHR of 2.1% during the first wave of COVID-19 (Menachemi et al.).

Importantly, we would also like to note that the specific choice of this parameter is not highly critical for the present analysis, since under the assumption of a constant infection-hospitalization proportion (which we discuss in more detail in the response to your comment

2.1) the estimated effective reproduction number is invariant to the absolute number of cases up to a scaling factor. Similarly, the simulated noise in infections and case counts will be comparable for hospitalization probabilities of the same order of magnitude. Hence, we tried to choose a proportion for our simulation that is compatible with estimates for COVID-19 in Switzerland, but we stress that our results are not dependent on the exact choice of this parameter.

References:

- Stringhini, S. et al. Seroprevalence of anti-SARS-CoV-2 IgG antibodies in Geneva, Switzerland (SEROCoV-POP): a population-based study. The Lancet 396, 313–319 (2020).
- Menachemi, N., Dixon, B. E., Wools-Kaloustian, K. K., Yiannoutsos, C. T. & Halverson, P. K. How Many SARS-CoV-2-Infected People Require Hospitalization? Using Random Sample Testing to Better Inform Preparedness Efforts. J Public Health Manag Pract 27, 246–250 (2021).

Comment 2.7: Line 292. It is unclear whether the data is simulated, analyzed and nowcasted daily or weekly, which is a very important aspect. Also the results plots (Figure 1-2) misses the y-axis label (e.g., Day by date of symptoms onset).

Response 2.7: Thank you for this suggestion, we have now clarified the various resolutions in the manuscript. The simulation has a daily resolution, nowcasts are conducted at selected points in time with daily resolution, and their performance is summarized at a weekly level. We have also added x-axis labels to all result figures.

Comment 2.8: Line 327-344. Missing reference/s for the WIS approach.

Response 2.8: We have added references to Bracher et al. (2021) and Gneiting and Raftery (2007).

Comment 2.9: Estimation of transmission variables from hospitalization records can be severely biased, particularly in the beginning of a pandemic and across different populations (Sherratt et al. 2021). Lines 371-376 could be brought up earlier in the subsection for clarity.

Response 2.9: We appreciate your suggestion and have added an explanation of the limitations of R_t estimation from hospitalization data early in the corresponding subsection.

References:

- Sherratt, Katharine, Sam Abbott, Sophie R. Meakin, Joel Hellewell, James D. Munday, Nikos Bosse, CMMID COVID-19 Working Group, Mark Jit, and Sebastian Funk. 2021. "Exploring Surveillance Data Biases When Estimating the Reproduction Number: With Insights into Subpopulation Transmission of COVID-19 in England." Philosophical Transactions of the Royal Society of London. Series B, Biological Sciences 376 (1829): 20200283.

Comment 2.10: While it is well detailed, it is difficult to follow the description of the differences between the approaches in the text, having to go back-and-forth to the figures. For clarity, I would recommend producing a table where the main, overall results (such as over- or under-estimation at different time-steps) are summarized.

Response 2.10: This is a good suggestion, we have added a summary table with the main findings to our Results section (see Table 1).

Comment 2.11: While formal comparison (based on WIS) shows better performance of the generative method in general, when applying the method in real world public health surveillance would require acknowledging the limitations of its performance. For example, while in Figure 2 first column row B and C, the generative method performs better, the nowcasted trend can be as misleading as that nowcasted by the stepwise approach from a public health perspective. This reviewers' interpretation of the overall results is that both methods somehow fail to provide reliable information, or at least, that the information that can be obtained in the very beginning of an outbreak with limited data is almost not useful for predicting transmission dynamics. Similar general interpretation can be made for R_eff estimates and when including missingness (Figures 3 , 4, 5). On the other hand, with downward trends and more data, the performance of both methods is reasonably good once they control for well known bias.

Response 2.11: We thank you for pointing out these important discussion points and agree that our manuscript has so far not sufficiently discussed the value of these nowcasting methods from a public health perspective. We also agree with your assessment that nowcasts very close to the present may not be adequately informed by observed data and therefore do not provide sufficient "information for action" regarding certain decisions in public health, at least not without additional considerations of changes in transmission etc. This is especially true for nowcasts of R_t, due to the additional delay by the incubation period. Nevertheless, if interpreted carefully, we believe that real-time estimates can contribute to situational awareness and short-term planning during an epidemic, and from this perspective, nowcasts can be seen as bridging the gap between retrospective estimation and plain forecasting. Here we argue that a generative approach has several desirable properties, namely faithful projection of initial growth dynamics and stationary R_t estimation, comprehensive uncertainty quantification, and higher interpretability and consistency (facilitating the integration of situational knowledge and additional data). However, we agree that the use of such nowcasts requires careful interpretation with respect to the public health question at hand. Therefore, in response to your comment, we have revised and expanded our Discussion section to better highlight these challenges (see expanded paragraph 3 and newly added paragraph 6 in Discussion).

Comment 2.12: The issues raised in the previous point also applied for the hospitalization data, with even more extreme trends (exponential trend nowcasted by generative method, over estimation etc)

Response 2.12: Yes, we agree, and the poor performance of nowcasts of both the stepwise and the generative approach especially in the first wave of the COVID-19 pandemic can likely be attributed to an inaccurate estimation of the reporting delay distribution, when reporting was

first established and then accelerated significantly (combined with overall limited data and abrupt changes in transmission). This both exacerbated the underprediction of the stepwise approach during the growth phase and the overprediction of the generative approach at the peak. To better highlight and explain these problems, we have revised our Discussion (see expanded paragraph 3 in Discussion section). We have also stressed the limitations of hospitalization data in our Methods section in reaction to your earlier comment 2.9 on R_t estimation from hospitalization records.

Comment 2.13: Line 565:. This somehow reflects the previous points. While the claim that the generative model performs statistically better in epidemic growth or decline is based on the WIS is true, from a surveillance perspective this might not necessarily be true, as the information that public health might be looking for (e.g., confirmation that the epidemic curve is stalling due to interventions) can not necessarily be obtained from the model nowcasts. Thus, while the method deals better with uncertainty and likely has better computational cost, the claim of a better performance, at least from a public health perspective (i.e., which information is useful) is arguable. Acknowledging the limitations of the generative method for its use in public health, not only from an statistical point of view, as acknowledging that of stepwise approaches, can improve the discussion on which methods are more useful and when can we use them reliably.

Response 2.13: Yes, this is a valid point, and we agree that the statistical performance of a method does not directly translate into its usefulness for public health. That is, for certain requirements, such as detecting changes in transmission as early as possible, the generative approach may not provide added value over existing approaches. In response to your feedback, we have therefore revised our Discussion to avoid general statements about the performance of our method and now provide a more detailed explanation of what we see as its strengths when used for situational awareness and short-term planning (see new paragraph 6 in Discussion, and our earlier reply to your comment 2.11). With these changes, we hope that our manuscript better recognizes the use of our nowcasting method from a public health perspective and provides helpful guidance on its interpretation to the reader. We thank you for your comments, which have helped us greatly in revising our manuscript in this regard.
